# Evolutionary dynamics of type VI secretion systems in fruit fly-associated *Enterobacter*

**DOI:** 10.3389/fmicb.2026.1755534

**Published:** 2026-03-05

**Authors:** Naima Bel Mokhtar, Panagiota Stathopoulou, Elias Asimakis, Antonios Augustinos, Julieta Salgueiro, Malini Alleck, Preeaduth Sookar, Óscar Dembilio, Diego F. Segura, George Tsiamis

**Affiliations:** 1 Laboratory of Systems Microbiology and Applied Genomics, Department of Sustainable Agriculture, University of Patras, Agrinio, Greece; 2 Department of Plant Protection Patras, Institute of Industrial and Forage Crops, Hellenic Agricultural Organization (DIMITRA), Patras, Greece; 3 Centro de Recursos Naturales Renovables de la Zona Semiárida, CERZOS (CONICET-Universidad Nacional del Sur), Buenos Aires, Argentina; 4 Agro-Industry and Food Security Division, Ministry of Agro-Industry, Food Security Blue Economy & Fisheries, Agricultural Services, Mauritius, Mauritius; 5 Empresa de Transformación Agraria S.A., S.M.E., M.P. (TRAGSA), Paterna, Spain; 6 Facultad de Ciencias Agronómicas y Veterinarias, Universidad del Salvador, Buenos Aires, Argentina; 7 Instituto de Genética “Ewald A. Favret” (INTA) —GV IABIMO (CONICET), Hurlingham, Argentina

**Keywords:** comparative genomics, fruit flies, microbiome, pangenomics, sit, symbiosis, Tephritidae

## Abstract

Species in the genus *Enterobacter* are widely distributed and occupy diverse ecological niches. Although many species within this genus have been extensively isolated and characterized, their symbiotic associations with Tephritidae fruit flies remain understudied, particularly through comparative genomic analyses. To address this gap, we conducted a whole-genome comparative analysis of thirteen *Enterobacter* strains isolated from the most economically significant fruit fly species: *Anastrepha fraterculus*, *Bactrocera dorsalis*, *Bactrocera zonata*, *Ceratitis capitata*, and *Zeugodacus cucurbitae*. The results revealed that different fruit flies harbor distinct *Enterobacter* species, with *Enterobacter hormaechei* being the most prevalent across hosts. Notably, distinct *E. hormaechei* subspecies were associated with specific hosts, suggesting a potential host-driven adaptation and coevolution. Pangenome analysis highlighted a dynamic genetic structure among these strains, with significant differences in the core, shell, and species-specific gene composition. The high proportion of metabolism-related genes in the core genome suggests a conserved role in essential biological functions, whereas the enrichment of mobile genetic elements (prophages and transposons) and cell motility genes within the shell and species-specific genomes highlights the genomic plasticity and potential host-specific adaptations. Three distinct subtypes of T6SS (type VI secretion systems) gene clusters, T6SS_C1, T6SS_C2, and T6SS_C3, were detected across *Enterobacter* strains. T6SS_C1 and T6SS_C2 were identified in most *Enterobacter* strains, whereas T6SS_C3 cluster was restricted to a single isolate. Although these clusters contained thirteen core T6SS genes, they were characterized by different gene synteny and effector/immunity gene content, suggesting that different *Enterobacter* strains may utilize distinct mechanisms for interbacterial interactions, host manipulation, and environmental adaptation. Overall, our findings reveal the genetic basis of the symbiosis between *Enterobacter* species and fruit flies, shedding light on their evolutionary dynamics, diversity of T6SS, and functional traits. These results open new avenues for developing microbiome-based strategies for pest management, including the targeted manipulation of microbial communities to enhance sterile insect technique (SIT) outcomes.

## 1 Introduction

Insects and their microbial symbionts form complex relationships that influence numerous biological functions within the host, including digestion, reproduction, and defense. These interactions play a crucial role in shaping the host’s physiology, ecology, and evolutionary processes (Mondal et al., 2023). The association between Tephritidae fruit flies (a family that includes several agriculturally devastating pests) and their bacterial symbionts is becoming increasingly important in both ecological and applied research (Noman et al., 2020). The Tephritidae family encompasses a diverse group of phytophagous species that infest a wide range of fruit and vegetable crops worldwide, causing severe economic losses through direct damage and quarantine restrictions (He et al., 2023). Many tephritid species depend on bacterial symbionts to survive in nutrient-poor or toxic plant tissues and to withstand environmental stress (Ben-Yosef et al., 2014; Cheng et al., 2017). These microbial partners, which often inhabit the insect gut or reproductive organs, play crucial roles in host survival, reproduction, and colonization of new environments.

The contribution of gut-associated bacteria is frequently nutritional, as they provide essential amino acids, vitamins, nitrogen, and carbon-based compounds, that help compensate for the nutrient-poor or imbalanced diets typical of both natural and mass-reared environments (Azis et al., 2019; Haytham et al., 2024). Other reported benefits include restoring gut microbiota imbalances caused by irradiation and mass-rearing conditions, as well as conferring resistance against pathogen proliferation or pesticides (Cai et al., 2018; Guerfali et al., 2021; Fan et al., 2025). Notably, members of the family Enterobacteriaceae are consistently reported as dominant and functionally important constituents of fruit fly gut communities. For instance, *Klebsiella oxytoca* has been shown to significantly reduce *Pseudomonas* spp. in *Ceratitis capitata*, highlighting the functional importance of native gut symbionts in host defense (Ben Ami et al., 2010).

Within the Enterobacteriaceae family, bacteria of the genus *Enterobacter* have gained attention as keystone players, often co-occurring with or performing analogous functions to *Klebsiella* within the insect gut. The *Enterobacter* genus includes a diverse group of facultative anaerobic Gram-negative bacteria (Grimont and Grimont, 2006). Members of this genus are ubiquitous in nature and commonly found in different ecological niches, including soil, plants, and insect gastrointestinal tracts (Jha et al., 2011; García-González et al., 2018; Wang et al., 2022; Siderius et al., 2024). Although some *Enterobacter* strains are opportunistic human pathogens associated with nosocomial infections and antibiotic resistance (Anju et al., 2020; Salimiyan Rizi et al., 2020), several other strains exhibit mutualistic roles, contributing to nutrient cycling (Ji et al., 2020), promoting plant growth (Kämpfer et al., 2005), and aiding insect digestion (Stathopoulou et al., 2021). This ecological versatility suggests a high degree of genome plasticity, which allows *Enterobacter* species to adapt to different hosts and environments.

The association between *Enterobacter* and fruit flies is particularly intriguing, as this bacterial genus has been characterized using classical microbiological and amplicon-based approaches as part of the core microbiome of various wild and laboratory-reared flies, including *Anastrepha fraterculus* (Salgueiro et al., 2020, 2022), *Bactrocera cacuminata* (Thaochan et al., 2010), *Bactrocera dorsalis* (Pramanik et al., 2014; Gujjar et al., 2017), *Bactrocera oleae* (Estes et al., 2012), *Bactrocera tryoni* (Thaochan et al., 2010), *Bactrocera zonata* (Naaz et al., 2020), *Ceratitis capitata* (Behar et al., 2008a; Morrow et al., 2015), *Rhagoletis pomonella* (Lauzon et al., 2000), and *Zeugodacus cucurbitae* (Asimakis et al., 2021; Hafsi et al., 2024). Notably, several culture-independent studies have reported *Enterobacter* as multiple operational taxonomic units or amplicon sequence variants, suggesting the coexistence of distinct species and potential subspecies of this genus within their host flies (Nikolouli et al., 2020; Asimakis et al., 2021; Salgueiro et al., 2022; Aoki et al., 2025). However, the limited taxonomic resolution of the 16S rRNA gene for discriminating closely related *Enterobacter* taxa constrains species- and strain-level identification. In this context, culture-based isolation is essential to resolve this knowledge gap, as it enables high-resolution taxonomic assignment, functional annotation, and experimental validation of host-microbe interactions (Augustinos et al., 2015; Hadapad et al., 2016; Kyritsis et al., 2019; Salgueiro et al., 2025). Extensive studies have linked *Enterobacter* strains to various host fitness traits, such as improved digestion, enhanced stress tolerance, and increased reproductive success (Augustinos et al., 2015). Due to these effects, fruit fly management increasingly integrates strategies that leverage symbiotic bacteria to enhance the efficiency of control methods, such as the Sterile Insect Technique (SIT) (Behar et al., 2008b; Sacchetti et al., 2014; Damodaram et al., 2016). For instance, *Enterobacter* sp. AA26 (Augustinos et al., 2015), isolated from *C. capitata*, is an effective nutritional supplement for both *C. capitata* and *B. oleae* larval diets under mass-rearing conditions. In particular, when incorporated “live” into the diet, *Enterobacter* sp. AA26 accelerates immature development of *C. capitata*, boosts pupal weight, improves adult recovery, and enhances survival under stressful conditions (Augustinos et al., 2015; Kyritsis et al., 2019; Koskinioti et al., 2020). Similarly, studies on *Z. cucurbitae* have reported positive outcomes when using both “live” and inactivated (autoclaved) *Enterobacter* spp. isolated from the gut, with inactivated bacteria providing marginally better enhancements in fitness parameters (Yao et al., 2017). In addition to nutrition, *Enterobacter* strains have been found to influence ecological interactions. For example, *Enterobacter cloacae* strains isolated from the guts of *B. dorsalis* and *B. zonata* and the feces of *R. pomonella* have been reported to strongly attract their respective hosts, allowing the attraction of additional flies to the infested fruit (Reddy et al., 2014; Duan et al., 2024). This offers an avenue for eco-friendly pest control using species-specific attractants.

Despite the taxonomic proximity of *Enterobacter* spp., the mechanisms underlying *Enterobacter*-fruit fly interactions appear to be species-specific, suggesting potential coevolutionary dynamics. However, the genetic basis of these interactions remains largely unexplored. A major limitation is the scarcity of *Enterobacter* genomes isolated from fruit flies, which hinders accurate classification and comparative studies. Comparative genomic analysis is a powerful tool for studying species-specific evolutionary processes, genomic rearrangements, genetic diversity, horizontal gene transfer events, and prophage-associated sequences (Land et al., 2015). Whole-genome sequencing (WGS) enables the analysis of pangenomes among different bacterial strains, including core, shared, and species-specific genes that define their main functional activities. Additionally, WGS facilitates the investigation of bacterial secretion systems, particularly the Type VI Secretion System (T6SS), which is a specialized mechanism that bacteria use to deliver effector proteins to neighboring cells (Cianfanelli et al., 2016; Singh and Kumari, 2023). T6SS plays a crucial role in interbacterial competition, host interactions, and adaptation to specific environments (Chen et al., 2015; LaCourse et al., 2018; Merciecca et al., 2022; Singh and Kumari, 2023). In *Enterobacter* spp., T6SS has been extensively studied in human-, healthcare-, and plant-associated strains (Soria-Bustos et al., 2020; Lucero et al., 2022; Anderson et al., 2023; Matte et al., 2024; Peng et al., 2024). However, their role in insects, particularly fruit flies, remains poorly understood.

To understand the variability of *Enterobacter* spp. associated with insects, this study performed a comparative genomic analysis of thirteen *Enterobacter* strains isolated from different populations of the most economically important fruit fly species: *A. fraterculus*, *B. dorsalis*, *B. zonata*, *C. capitata*, and *Z. cucurbitae*. With a focus on cultured isolates, this study enables genomic and functional analysis at the strain level, complementing the insights gained from culture-independent approaches. To the best of our knowledge, this is the first comprehensive genomic exploration of *Enterobacter* strains associated with fruit flies. By unraveling the microbial pangenome, strain-specific gene content, and functional features, such as T6SS, we aimed to decode the genetic basis of this symbiosis. These findings will contribute to future research on *Enterobacter*-fruit fly interactions and provide a framework for developing targeted strategies to enhance pest control.

## 2 Materials and methods

### 2.1 Sample collection and bacterial strain isolation

*Enterobacter* spp. used in this study were isolated from various fruit fly populations. Flies, including *A. fraterculus* (Af), *B. dorsalis* (Bd), *B. zonata* (Bz), *C. capitata* (Cc), and *Z. cucurbitae* (Zc), were collected from both laboratory and wild sources across different countries (Table 1). The flies were surface-sterilized with 70% ethanol and washed with sterile 1% phosphate-buffered saline (PBS) solution. Three to five adult male and female flies were pooled and homogenized in a sterile 1% PBS solution. Three replicates were prepared for each homogenate. The homogenate was serially diluted, and 100 μL were plated on Luria-Bertani (LB) agar medium [composed of 1% (wt/vol) peptone, 1% (wt/vol) NaCl, 0.5% (wt/vol) yeast extract, and 1.5% (wt/vol) agar] and incubated at 25, 30, and 37°C under aerobic conditions. Forty to 50 morphologically distinct colonies were picked from each host and underwent molecular identification.

**TABLE 1:** *Enterobacter* strains isolated in this study.

Genome_ID	Host	Sample type	Origin	Sequencing method
Af_WL114	*A. fraterculus*	LAB	Buenos Aires, Argentina	Illumina
Af_WL315	*A. fraterculus*	LAB	Buenos Aires, Argentina	Illumina
Bd_23A	*B. dorsalis*	LAB	South Africa	MinION
Bz_SP194	*B. zonata*	LAB	Mauritius	Illumina
Bz_SP209	*B. zonata*	WILD	Mauritius	Illumina
Bz_SP204	*B. zonata*	LAB	Mauritius	Illumina, MinION
Bz_SP308	*B. zonata*	WILD	Mauritius	Illumina, MinION
Cc_172122	*C. capitata*	LAB	Valencia, Spain	Illumina
Cc_172123	*C. capitata*	LAB	Valencia, Spain	Illumina
Cc_173601	*C. capitata*	LAB	Valencia, Spain	Illumina, MinION
Zc_SP163	*Z. cucurbitae*	WILD	Mauritius	Illumina
Zc_SP178	*Z. cucurbitae*	WILD	Mauritius	Illumina, MinION
Zc_SP171	*Z. cucurbitae*	WILD	Mauritius	MinION

### 2.2 Molecular identification of *Enterobacter* strains

Colony PCR (Woodman et al., 2016) was performed on single bacterial colonies using a KAPA Taq Polymerase kit (KAPA BioSystems) and universal 16S rRNA gene primers 27F/1492R (Weisburg et al., 1991) according to the manufacturer’s recommendations. The PCR cycling conditions include an initial denaturation at 95°C for 5 min, followed by 35 cycles of denaturation at 95°C for 30 s, annealing at 55°C for 1 min, extension at 72°C for 2 min, and a final elongation at 72°C for 10 min. The PCR products were run on a 1.5% (w/v) agarose gel in TBE buffer, and the positive PCR products were then precipitated using polyethylene glycol (20% PEG, 2.5 M NaCl) (Hartley and Bowen, 1996). Sanger sequencing was performed on purified positive PCR products with the BigDye Terminator v3.1 Cycle Sequencing Kit, following the manufacturer’s recommendations (Applied Biosystems, Foster City, CA, United States). Reaction products were purified using an ethanol/EDTA protocol following the manufacturer’s instructions (Applied Biosystems) and analyzed on an ABI PRISM 3,500 Genetic Analyzer (Applied Biosystems). The 16S rRNA gene consensus reads were generated from forward and reverse sequences and compared with the type strains of known bacterial species using BLAST.^1^ Among the isolated strains, all thirteen strains identified as belonging to the genus *Enterobacter* were subjected to whole-genome sequencing (Table 1). The strains were labeled using the host abbreviation followed by the strain code (e.g., Cc_17601 represents *Enterobacter* strain 17,601 isolated from *C. capitata*) (Table 1).

### 2.3 Whole genome sequencing

Genomic DNA was extracted from a single colony using a modified cetyl-trimethylammonium bromide (CTAB) protocol (Doyle and Doyle, 1990). A combination of short- and long-read sequencing was employed to sequence the isolated strains. For strains in which Illumina sequencing produced low-quality data (or yielded low-quality reads), ONT sequencing was used to generate complete or near-complete genome assemblies. The sequencing technologies used for each strain are detailed in Table 2. For Illumina sequencing, the genomic DNA library was prepared using the Nextera XT Library Prep Kit (Illumina, San Diego, CA, United States), following the manufacturer’s protocol. The library was quantified using the KAPA Biosystems Library Quantification Kit for Illumina on a Roche Light Cycler 96 qPCR machine and sequenced with a 75X coverage on the Illumina HiSeq 2,500 using a 250 bp paired-end protocol (MicrobesNG, Birmingham, United Kingdom). High-molecular-weight genomic DNA from the selected strains was sequenced on a MinION device (Oxford Nanopore Technologies) using the genomic DNA sequencing kit SQK-LSK114, according to Oxford Nanopore Technologies’ instructions. The libraries were quantified using the Qubit™ dsDNA HS Assay kit by fluorescence measurement. Purified, adapter-ligated DNA was sequenced on an MK1B (MIN-101B) MinION platform with an R10.4.1 Flongle (FLO-FLG114) using MinKNOW software v2.10. After sequencing, POD5 files were base-called using Guppy v3.1.5. The sequencing reads for all *Enterobacter* spp. isolates are available in NCBI under BioProject accession number PRJNA1093357.

**TABLE 2:** General features of the genomes of the thirteen *Enterobacter* strains examined in this study.

GenomeID	Host	Assembly quality						Completeness (%)		Genomic features			
		Contigs	Largest contig	Total length	GC (%)	N50	L50	CheckM	Busco	CDS	rRNA	tRNA	Repeat region
Af_WL114	*A. fraterculus*	668	51,907	4,477,744	55.54	10,471	130	99.3	99.2	4,338	3	26	0
Af_WL315	*A. fraterculus*	59	482,717	4,580,003	55.23	167,795	9	99.97	99.2	4,354	5	74	0
Bz_SP194	*B. zonata*	44	467,653	4,657,560	56.07	224,772	8	99.07	99.2	4,384	10	68	0
Bz_SP209	*B. zonata*	27	889,036	4,699,445	55.57	339,326	5	99.47	98.4	4,452	10	65	0
Cc_172122	*C. capitata*	67	620,825	4,904,022	55.23	226,393	6	99.77	98.4	4,831	6	67	0
Cc_172123	*C. capitata*	72	638,596	4,892,600	55.23	315,136	5	99.77	98.4	4,821	2	69	0
Zc_SP163	*Z. cucurbitae*	36	1,221,988	5,129,040	55.13	235,287	6	99.97	99.2	4,945	5	69	0
Bz_SP204	*B. zonata*	2	4,744,279	4,760,161	55.96	4,744,279	1	99.07	99.2	4,458	25	87	24
Bz_SP308	*B. zonata*	1	4,630,003	4,630,003	55.68	4,630,003	1	99.47	98.4	43,031	25	83	31
Cc_173601	*C. capitata*	4	4,728,438	4,927,162	55.68	4,728,438	1	99.97	99.2	4,708	25	87	43
Zc_SP178	*Z. cucurbitae*	3	4,913,762	5,175,407	55.12	4,913,762	1	99.97	99.2	4,944	25	84	48
Bd_23A	*B. dorsalis*	6	4,946,180	5,471,282	55.07	4,946,180	1	99.02	96.8	5,538	25	83	69
Zc_SP171	*Z. cucurbitae*	4	4,780,983	5,334,018	53.07	4,780,983	1	98.41	97.6	5,546	25	86	75

### 2.4 Genome assembly, annotation, and classification

The resulting reads were assessed and filtered for quality. Trimmomatic (Bolger et al., 2014) was applied to short reads with criteria set at Qscore > 20 and read length > 200 bp, while NanoFilt (De Coster et al., 2018) was used for long reads with criteria set at Qscore > 10 and read length > 1,000 bp. *De novo* assembly of Illumina-based sequencing reads was performed using the Shovill v1.1.0 assembler^2^ with default parameters. High-quality ONT sequencing reads were *de novo* assembled using Flye v2.9.2 (Kolmogorov et al., 2019) followed by a single round of polishing using Medaka v1.11.3.^3^ For hybrid *de novo* assembly, the initial assembly of long reads was conducted using Flye v2.9.2 (Kolmogorov et al., 2019). The resulting contigs were first polished using Medaka v1.11.3 (see text footnote 3), followed by Polypolish v0.5.0 (Wick and Holt, 2022) with short read sequences. The quality of the resulting assemblies was evaluated using Quast (Gurevich et al., 2013). CheckM (Parks et al., 2015) and BUSCO (Simão et al., 2015) were used to assess genome completeness based on default parameters. All assembled genomes were annotated using RAST toolkit (Overbeek et al., 2014; Brettin et al., 2015). Taxonomic classification based on the assembled genomes was performed using GTDB-Tk (Chaumeil et al., 2020) against the Genome Taxonomy Database Taxonomy (GTDB) release 09-RS220 (Parks et al., 2022). Pairwise average nucleotide identity (ANI) and amino acid identity (AAI) between *Enterobacter* spp. genomes and reference bacterial species were determined using FastANI (Jain et al., 2018) and EzAAI (Kim et al., 2021), respectively. To refine the phylogenomic classification at the subspecies level, we conducted an additional comparison between the genomes generated in this study and 56 publicly available genomes in the NCBI database (Supplementary Table 1). Reference genomes corresponding to type strains and representative genomes were included when available. Additional complete and scaffold genomes were retrieved based on the assembly quality, including high completeness and low contamination. Additional subspecies-level characterization was performed by submitting the genome sequence data to the Type (Strain) Genome Server (TYGS),^4^ which estimates digital DNA-DNA hybridization (dDDH) values and confidence intervals via Genome-to-Genome Distance Calculator (GGDC) 4.0 using the recommended settings (Meier-Kolthoff et al., 2013; Meier-Kolthoff and Göker, 2019). A phylogenomic tree based on whole-genome sequences was constructed using Mash, a Kmer-based approach (Katz et al., 2019), and customized using the iTOL online tool (Letunic and Bork, 2021).

### 2.5 Host and *Enterobacter hormaechei* coevolution

To assess the association between the host and *E. hormaehei* subspecies, a cophylogenetic analysis was conducted based on their respective phylogenetic trees. The host phylogeny was inferred using publicly available COI, COII, ND1, ND2, ND3, ND4, and ND5 gene sequences. The sequences of each gene were aligned using ClustalW (Thompson et al., 1994), and phylogeny was constructed using BEAST (Bouckaert et al., 2014) with default parameters. The *E. hormaechei* subspecies phylogenetic tree was constructed using “Codon Trees” on the BV-BRC platform v3.42.3 based on 500 single-copy gene families selected from the *E. hormaechei* subspecies genomes: Af_WL114, Af_WL315, CC_172122, CC_172123, Bz_SP308, Bz_SP209, and Bd_23A. Cophylogenetic analysis was conducted using distance-based methods ParaFit (Legendre et al., 2002) and PACo (Balbuena et al., 2013), as implemented in APE (Paradis et al., 2004) and paco (Hutchinson et al., 2017) R packages, respectively. ParaFit and PACo assess the congruence between the host and *Enterobacter* strains (symbiont) phylogenetic distance mediated by the matrix of host-*Enterobacter* links.

### 2.6 Pangenome analysis

The pangenome analysis of all the isolated *Enterobacter* species was performed using Roary v3.13.0 (Page et al., 2015) based on modified GFF3 files generated by RAST annotation. Given the close relatedness of the *Enterobacter* species, the BLASTp percentage identity threshold in Roary was set to 90%. The Af_WL114 strain was excluded from the analysis due to the high fragmentation of its genome assembly (Table 2). The identified orthologous clusters among genomes were classified into three categories: Core, which includes genes that are present in all the analyzed strains; Shell, which includes genes found in more than one species; and Species-specific, which consists of genes found in at least one strain of the same species. The resulting alignment of concatenated core genes was then used for the construction of a neighbor-joining phylogenetic tree using Geneious.^5^ Functional annotation of the identified clusters was performed by comparing the representative sequences of each gene cluster against the updated 2024 Clusters of Orthologous Genes database (COGs) (Tatusov et al., 2000) using COGclassifier.^6^ Fisher’s Exact Test (Benjamini-Hochberg correction, with an error rate of 0.05) was used to confirm the statistical enrichment of the assigned functions between the pangenome categories. For example, for the COG category “U” in the core genome, Fischer’s exact test was applied to core vs. non-core gene clusters (which includes shell + species-specific gene clusters) and COG category “U” vs. non-COG category “U” gene clusters. Statistical analysis and visualization of the pangenome data were conducted using R v4.1.2.

### 2.7 Identification and classification of Type VI secretion systems

To elucidate the mechanisms by which *Enterobacter* spp. interact with their fruit fly hosts and other bacterial members of their community, the T6SS was analyzed, as it plays a key role in mediating bacterial competition, virulence, and host adaptation through effector protein delivery. T6SS clusters in *Enterobacter* strains were identified using MacSyFinder (Abby et al., 2014) based on TXSScan model (Abby and Rocha, 2017) using default settings. A BLASTp search was also used to examine putative effectors and immunity protein genes inserted within or around the T6SS core components. The classification of the identified T6SS clusters was based on the TssB protein, as it has been suggested to be a reliable marker for the classification (Barret et al., 2011; Russell et al., 2014). TssB sequences were extracted from the T6SSs clusters and submitted to the SecReT6 web platform^7^ for classification using the T6SS classification tool (Li et al., 2015). The TssB phylogeny encompassed the 26 TssB sequences identified across the *Enterobacter* strains in this study, along with the experimentally validated TssB proteins recorded in SecReT6. Initially, TssB sequences were aligned using MAFFT v7.475 (Katoh and Standley, 2013). A phylogenetic tree was then constructed using FastTree v2.1.11 (Price et al., 2009) and customized using the iTOL online tool (Letunic and Bork, 2021). The genetic organization of the secretion systems among the investigated genomes was visualized using the Python package pygenomeviz v0.4.4.^8^ Homologous coding sequences (CDS) between T6SS clusters were clustered using MMseqs2 (Steinegger and Söding, 2017).

## 3 Results

### 3.1 General features of *Enterobacter* spp. genomes

To characterize the genomic features of *Enterobacter* spp. strains isolated from fruit flies, 13 isolates were sequenced and *de novo* assembled. The genome sizes ranged from 4.57 to 5.57 Mbp, with GC content varying between 53.07 and 56.07% (Table 2). A hybrid approach was used for the genome assembly of four strains, combining short Illumina reads with long Oxford Nanopore (ONT) reads (Bz_SP204, Bz_SP308, Cc_173601, and Zc_SP178). Only the long ONT reads were used for two strains (Bd_23A and Zc_SP171). Hybrid and ONT-based approaches resulted in highly contiguous assemblies, with contig counts ranging from one to six and N50 lengths between 4,913,762 and 4,946,180 bp. The remaining seven strains were assembled using the short-read Illumina approach, resulting in draft genomes with contig counts between 27 and 668 bp and N50 lengths ranging from 10,471 to 339,326 bp. The assembled genomes exhibited high completeness, ranging from 98.41 to 99.97% according to CheckM and from 96.80 to 99.20% according to BUSCO. The number of coding sequences (CDS), as determined using the RAST annotation pipeline, ranged from 4,331 in Bz_SP308 to 5,546 in Zc_SP171. Wide variations in rRNA and tRNA copy numbers were observed, with genomes sequenced using hybrid approaches or ONT alone exhibiting higher copy numbers. General features of the sequenced genomes are listed in Table 2.

### 3.2 Whole genome-based classification of *Enterobacter* spp. strains

**FIGURE 1:**
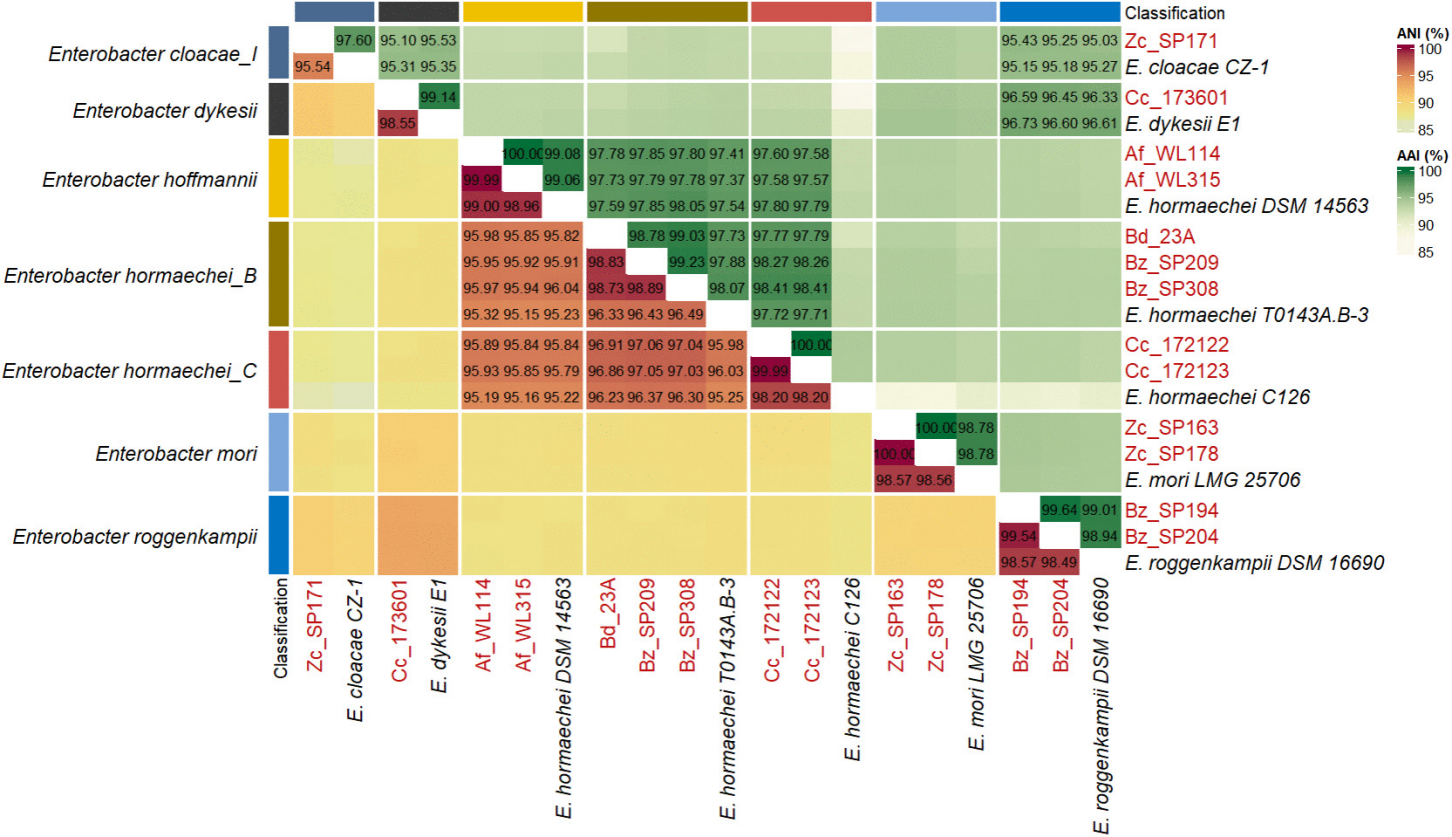
Average Nucleotide Identity (ANI) and Average Amino Acid Identity (AAI) from whole-genome comparison. *Enterobacter* isolated in this study (red) and their closest relatives from GTDB release 220 (black). ANI and AAI values were estimated using the complete genome assemblies and protein-coding genes, respectively.

The genomes of the *Enterobacter* strains isolated from different fruit fly species were classified based on the GTDB release 220, revealing seven distinct groups corresponding to six different species: *Enterobacter roggenkampii* (Bz_SP194 and Bz_204), *Enterobacter dykesii* (Cc_173601), *Enterobacter mori* (Zc_SP163 and Zc_SP178), *Enterobacter cloacae_I* (Zc_SP171), *Enterobacter hoffmannii* (Af_WL114 and Af_WL315) along with five strains classified as two distinct subspecies of *Enterobacter hormaechei*; *Enterobacter hormaechei_B* (Bd_23A, Bz_SP209, and Bz_SP308) and *Enterobacter hormaechei_C* (Cc_172122 and Cc_172123) (Supplementary Table 2 and Figure 1). Within the GTDB, ANI and AAI values between the three genomes representing *E. hormaechei_B* (*E. hormaechei* T0143A.B-3; GCF_008082005.1), *E. hormaechei_C* (*E. hormaechei* C126; GCA_006385655.1), and *E. hoffmannii* (*E. hormaechei* DSM 14563; GCF_001729745.1) were consistent with their classification as distinct subspecies (Rosselló-Móra and Amann, 2015). Notably, the *E. hoffmannii* group represented by the type strain DSM 14,563 was classified differently across databases. According to NCBI, it is identified as *E. hormaechei* subsp. *hoffmannii*, whereas GTDB classifies it under the *Enterobacter hoffmannii* clade, aligning with studies that suggest that *Enterobacter hoffmannii* is a distinct species rather than a subspecies of *E. hormaechei* (Wu et al., 2020). However, since most *Enterobacter hoffmannii* strains were initially deposited as *E. hormaechei* subsp. *hoffmannii*, the NCBI taxonomy will be adopted for consistency throughout this manuscript.

**FIGURE 2:**
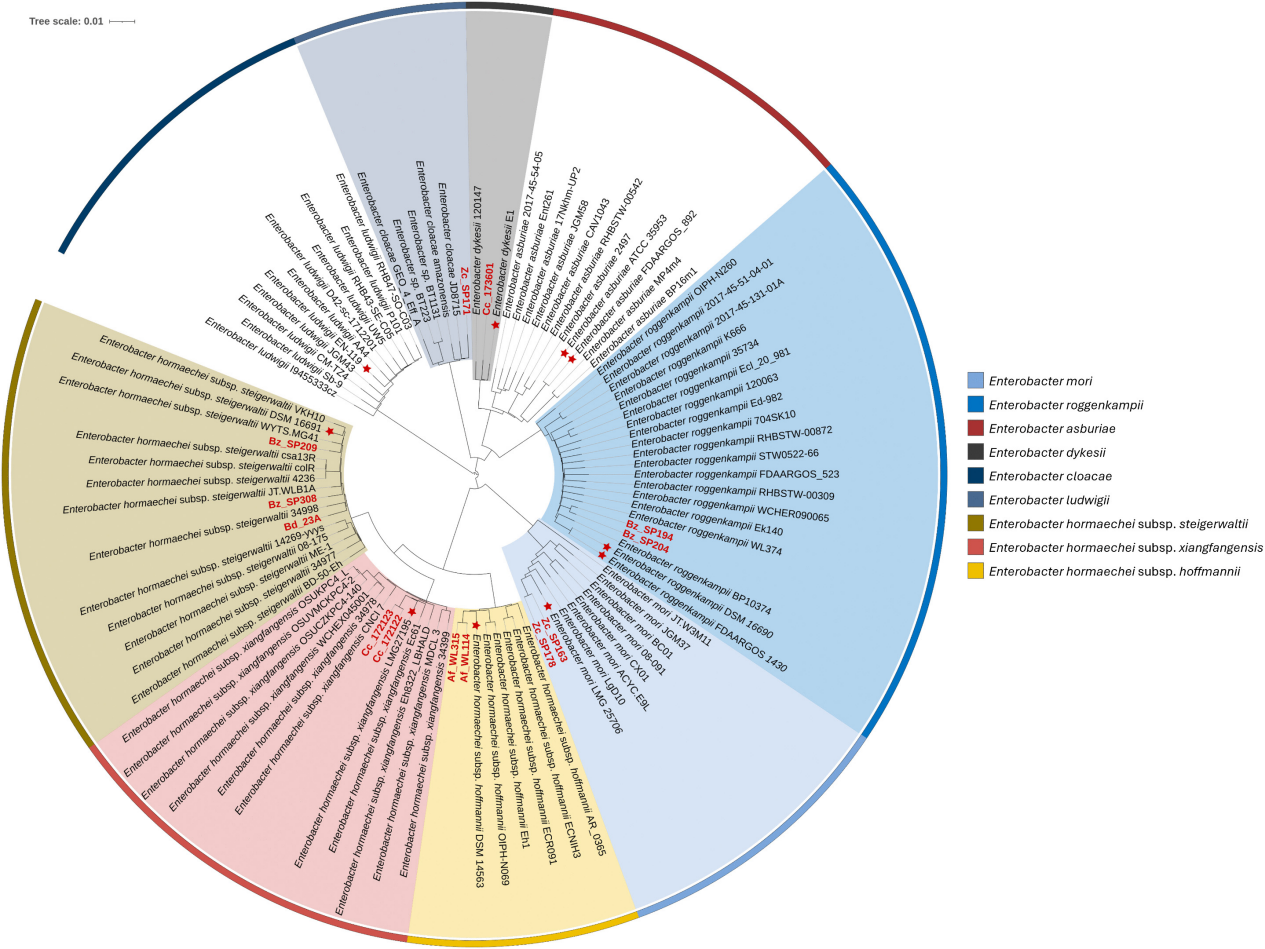
Phylogenetic placement of *Enterobacter* isolates. Distribution of *Enterobacter* strains isolated in this study (red labels) with closely related reference genomes of *Enterobacter* species and subspecies. Type strains were indicated with red asterisks (*). Phylogenetic distances were estimated using Mash, a kmer-based approach, as implemented in Mashtree software. Tree visualization was performed on ITOL online tool.

Despite their classification within *E. hormaechei_B*, the strains Bd_23A, Bz_SP209, and Bz_SP308 showed slightly higher ANI values compared to the representative genome of *E. hormaechei_C* (Figure 1). To clarify the relationships within the genomes classified as *E. hormaechei* and to accurately identify the various subspecies, a set of complete or scaffolded genomes was acquired from publicly available data in the NCBI database. This dataset included type strains from different *E. hormaechei* subspecies, including *E. hormaechei* subsp. *hormaechei*, *E. hormaechei* subsp. *oharae*, *E. hormaechei* subsp. *steigerwaltii*, *E. hormaechei* subsp. *xiangfangensis*, and *E. hormaechei* subsp. *hoffmannii* (Supplementary Table 1). Phylogenetic analysis based on whole-genome sequences using the Mashtree algorithm showed evidence of subspecies classification within the *E. hormaehei* clade (Figure 2). The pairwise ANI and dDDH analyses confirmed that the strains Bd_23A, Bz_SP209, and Bz_SP308 belonged to *E. hormaechei* subsp. *steigerwaltii*, the strains Cc_172122 and Cc_172123 to *E. hormaechei* subsp. *xiangfangensis* and the strains Af_WL114 and Af_WL315 to *E. hormaechei* subsp. *hoffmannii*. Notably, each of these strains exhibited an ANI value above 98% and a dDDH value above 91% relative to other reference genomes within the same subspecies, confirming their taxonomic assignment with high confidence (Supplementary Figure 1 and Supplementary Table 3). Using GTDB release 220, the representative genome of *E. hormaechei_B* clade (*E. hormaechei T0143A.B-3*, GCF_008082005.1) showed a lower ANI value (96.32–96.49%) relative to the genomes of *E. hormaechei* subsp. *steigerwaltii* (Supplementary Figure 1). As a verification step, in the updated GTDB release 226, the *E. hormaechei_B* and *E. hormaechei_C* clades were merged under a single representative genome. The updated analysis of the strain isolated in this study against the latest release confirmed the taxonomic classification of the strains (Supplementary Table 3).

### 3.3 Potential evidence of host-*Enterobacter* subspecies coevolution

**FIGURE 3:**
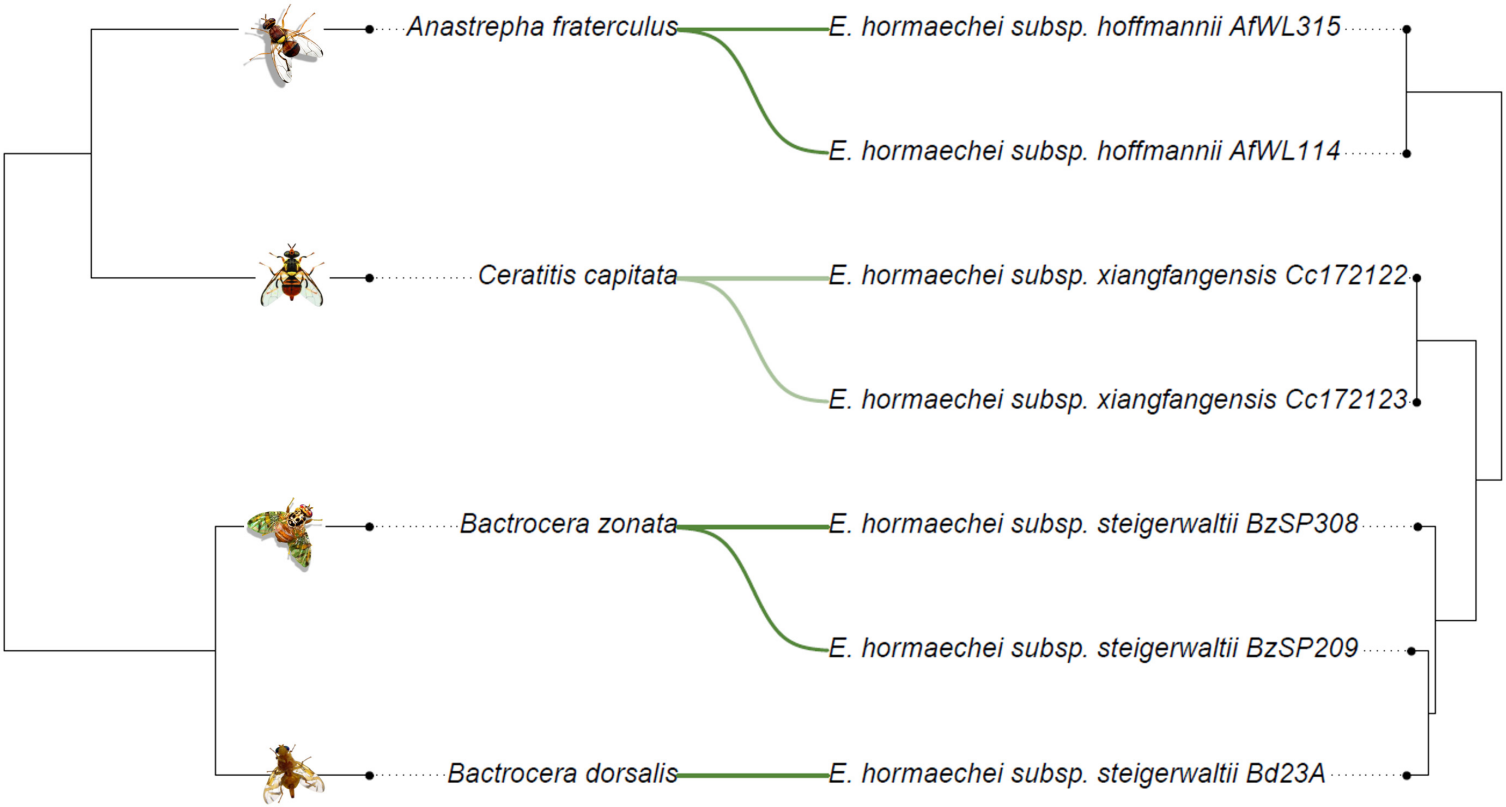
Tanglegram of cophylogenetic relationship between fruit fly hosts and *Enterobacter hormaechei* subspecies. *Enterobacter* spp. phylogeny was constructed based on whole genome assembly and host phylogeny inferred using COI, COII, ND2, ND4, and ND5 genes (left). Solid green lines indicate significant host-*Enterobacter* associations supported by both ParaFit and PACo analyses, while light green lines represent associations that are significant only with PACo.

The phylogenetic relationships among the *E. hormaechei* subspecies, including the recently described *E. hoffmannii*, appear to partially reflect the evolutionary history of their respective fruit fly hosts (Figure 3). *Anastrepha fraterculus* and *C. capitata* formed a distinct clade separate from *B. zonata* and *B. dorsalis* (Figure 3). Similarly, within *E. hormaechei*, subspecies-level associations were non-exclusive, with *E. hormaechei* subsp. *steigerwaltii* detected in more than one host species, *B. dorsalis* and *B. zonata* (Figure 3). The observed host-*E. hormaechei* associations were supported by cophylogenetic analyses rather than host-specificity, as evidenced by ParaFit and PACo analyses. Both analyses revealed statistically significant evidence of coevolution between *E. hormaechei* subspecies and their hosts (global test: *P*-value < 0.002). Pairwise tests of host-*Enterobacter* associations were significant using both methods (Supplementary Table 4). However, the cophylogenetic associations between *C. capitata* and *E. hormaechei* subsp. *xiangfangensis* strains were significant using one of the two methods, suggesting that these strains may not exhibit a strong host-specific relationship (Figure 3 and Supplementary Table 4).

### 3.4 Insights into the pangenome composition of *Enterobacter* spp.

**FIGURE 4:**
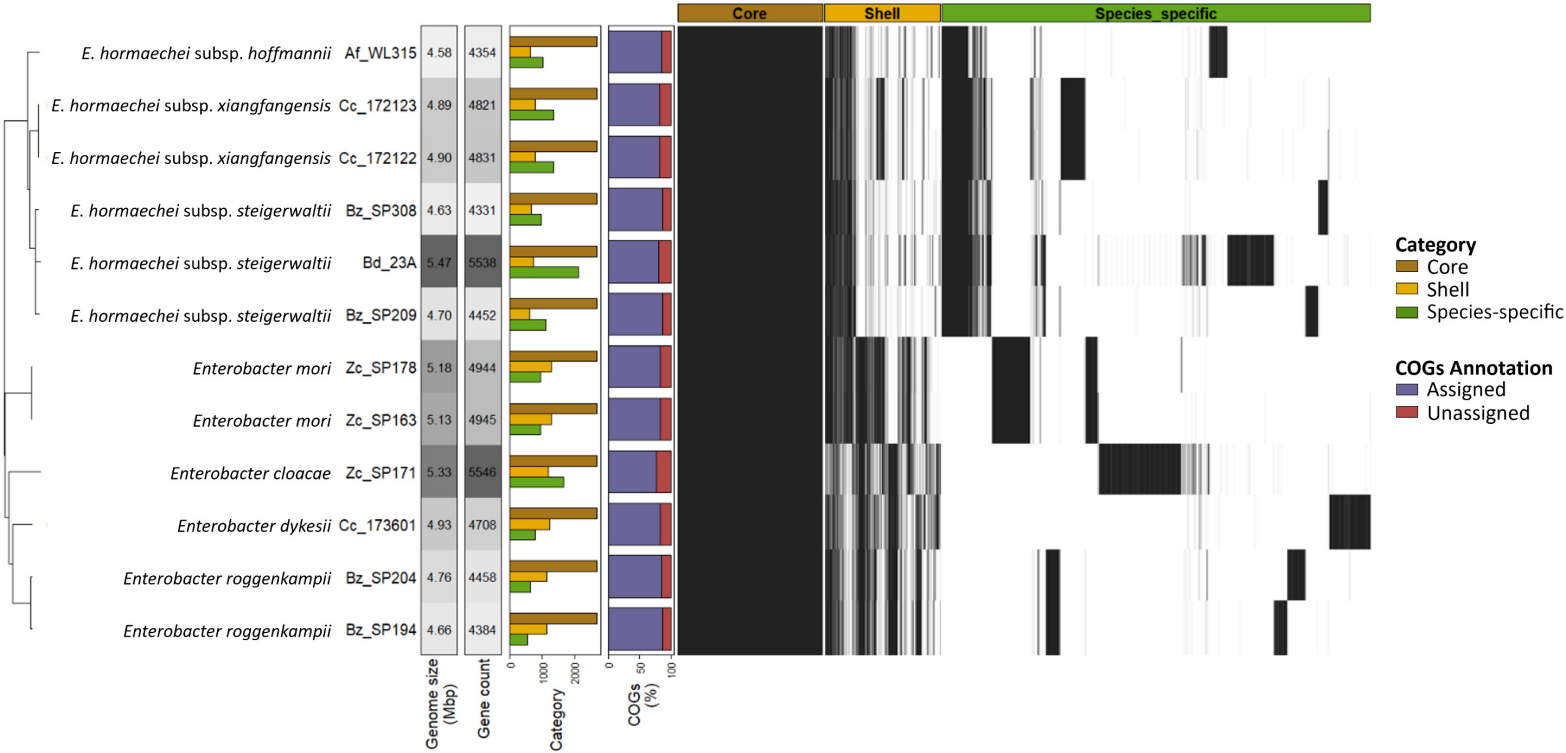
Pangenome analysis of *Enterobacter* species. From left: Phylogenetic tree based on core genes. The genome size and gene count were estimated using QUAST and RAST annotation, respectively. The genes per isolate were grouped into core, shell, and species-specific categories. Percentage of Assigned/Unassigned Clusters of Orthologous Genes (COGs) per isolate. Heatmap of gene presence/absence per isolate.

All *Enterobacter* strains isolated in this study were included in the pangenome analysis except for strain Af_WL114, which was excluded due to the highly fragmented state of its genome assembly. The resulting pangenome consisted of 12,789 orthologous clusters, including 2,696 core genome clusters (21.10%), 2,144 shell genome clusters (16.76%), and 7,949 species-specific genomes (62.15%) (Figure 4), indicating significant genetic diversity among strains. The number of shell clusters varied notably, from 626 clusters in *E. hormaechei* subsp. *steigerwaltii* (Bz_SP209) to 1,289 clusters in *E. mori* (Zc_SP178). Interestingly, the distribution of species-specific clusters showed that *E. cloacae* (Zc_SP171) (1,667 clusters) had the highest number of gene clusters, followed by *E. hormaechei* (average 1,323 clusters). In contrast, *E. roggenkampii* strains (Bz_SP204 and Bz_SP194) have the lowest number of gene clusters (average 587 clusters) (Figure 4 and Supplementary Figure 2A). At the strain-specific level, *E. hormaechei* subsp. *xiangfangensis* strains (Cc_172122 and Cc_172123) and *E. mori* (Zc_SP178 and Zc_SP163) had the lowest number of unique gene clusters (< 40 genes) and a high number of shared clusters (538 and 941 clusters, respectively) (Supplementary Figure 2A). The combination of a high number of shared gene clusters and a low number of unique gene clusters suggests that most of these shell genes are specific to *E. hormaechei* subsp. *xiangfangensis* and *E. mori*, respectively.

**FIGURE 5:**
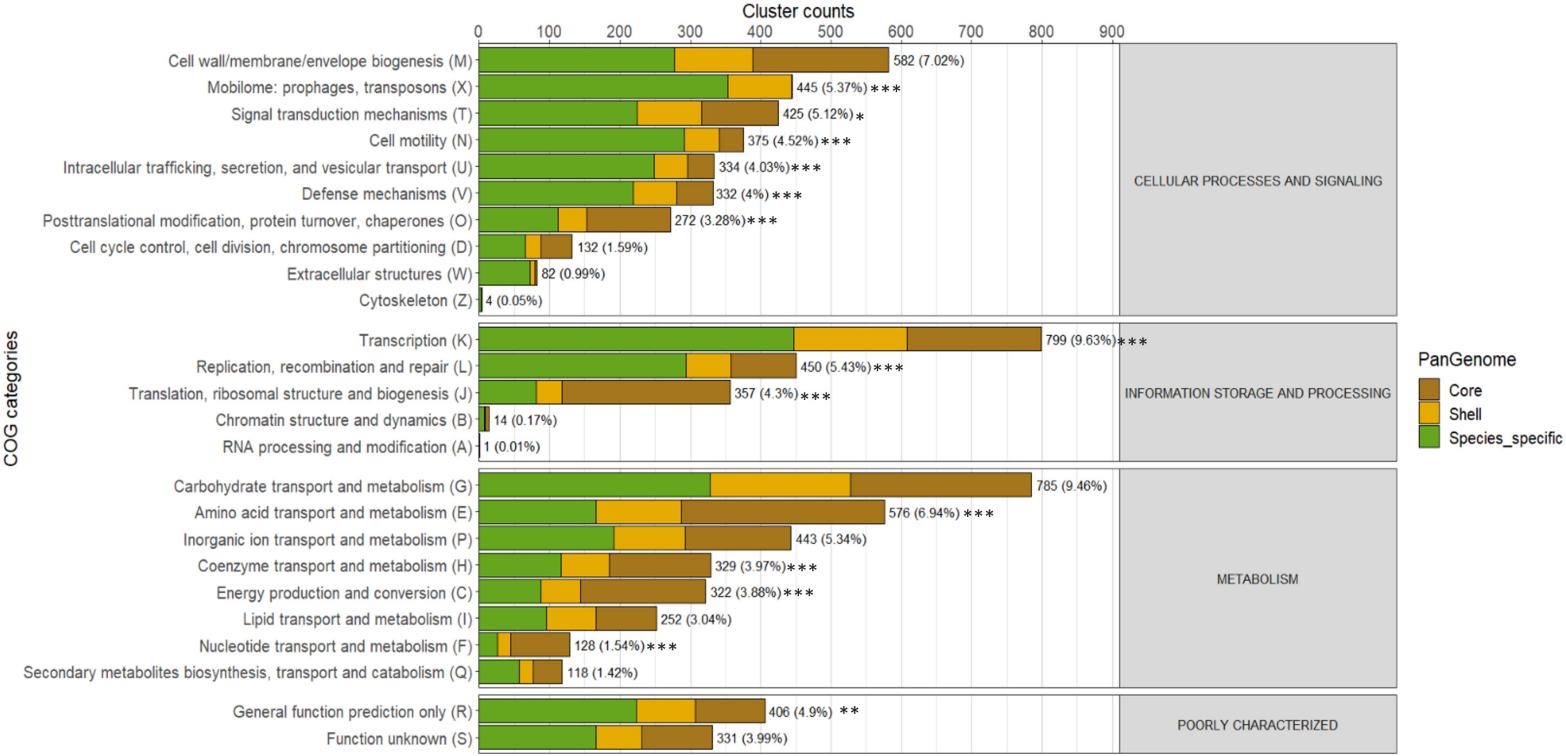
Distribution of COGs functional categories across *Enterobacter* pangenomes. The numbers and percentages in each bar chart represent the total gene family counts assigned to each COG category and their proportions. Asterisk (*) indicates the statistical significance based on Fisher’s exact test for the differential presence of a specific COG category between core and accessory genes (shell + species-specific). Significance levels are indicated as follows: *P < 0.05, **P < 0.01, and ***P < 0.001 (Benjamini-Hochberg corrected *P*-values).

A total of 8,294 orthologous clusters (64.8%) were annotated within the Cluster of Orthologous Groups (COGs) of protein functional categories. A significant portion of the core (2,548, 94.5%) and shell genomes (1,594, 74.3%) were assigned to functional categories. In contrast, only 4,152 species-specific clusters (52.2%) were annotated (Supplementary Figure 2B). Core and shell genomes were enriched with groups related to Metabolism (48.2 and 41.3%, respectively; Fisher’s exact test, *P* < 0.001), whereas species-specific genomes were highly enriched with cellular processing and signaling (45.0%, respectively; Fisher’s exact test, *P* < 0.001) (Supplementary Figure 2C). The core genome was significantly enriched with COGs contributing to basic vital functions, including “Amino acid transport and metabolism (E),” “Energy production and conversion (C),” and “Translation, ribosomal structure and biogenesis (J)” (Fisher’s exact test, *P* < 0.05) (Figure 5 and Supplementary Table 5). Although various COG categories were detected within the shell genome, none exhibited significant enrichment. However, the species-specific genome appears to include various COG categories, which were significantly enriched in functions related to genome plasticity. Notably, “Mobilome: prophages, transposons (X)” (353 gene clusters; Fisher’s exact test, *P* < 0.001) were enriched in *E. cloacae*, *E. dykesii*, and *E. hormaechei*. “Cell motility (N)” (292 gene clusters; Fisher’s exact test, *P* < 0.001) was mainly present in *E. hormaechei*, and “Coenzyme transport and metabolism (H)” (116 gene clusters; Fisher’s exact test, *P* < 0.001) in *E. cloacae* (Figure 5 and Supplementary Figure 3). Other categories, such as “Intracellular trafficking, secretion, and vesicular transport (U)” and “Defense mechanisms (V),” were present with less variation across species (249 and 119 clusters, respectively) (Figure 5 and Supplementary Figure 3).

Within the species-specific genome, the “Mobilome: prophages, transposons (X)” category was enriched in *E. cloacae* (76 genes) and *E. hormaechei* species, including *E. hormaechei* subsp. *steigerwaltii* (Bd_23A) with 108 genes, and *E. hormaechei* subsp. *xiangfangensis* (and Cc_172122 and Cc_172123) with 68 and 67 genes, respectively. While the strains Bz_SP209 and Bz_SP308 (*E. hormaechei* subsp. *steigerwaltii*) showed minimal content (7 genes each) (Supplementary Figure 4). A detailed investigation of functional annotation revealed 21 phage-related gene families distributed across 230 genes, primarily shared among *E. hormaechei* species, mainly between *E. hormaechei* subsp. *xiangfangensis* (and Cc_172122 and Cc_172123) (47 and 46 genes, respectively) followed by *E. cloacae* (Zc_SP171) (34 genes). Additionally, 10 transposase-related families distributed across 176 genes were identified, most of which were uniquely identified within the *E. hormaechei* subsp. *steigerwaltii* (Bd_23A) strain (72 genes) (Supplementary Figure 4). Genes associated with “Cell motility (N)” category, including type VI pili, type II secretion system, and flagellar-related genes, were found across all genomes. However, more species-specific genes were identified among *E. hormaechei* genomes, mainly in *E. hormaechei* subsp. *steigerwaltii* (Bd_23A and Bz_SP209) (91 and 85 genes, respectively) (Supplementary Figure 5). The “Coenzyme transport and metabolism (H)” category contained numerous core genes. Notably, 20 genes involved in Cobalamine (Vit. B12) biosynthesis were exclusive to *E. cloacae* strain Zc_SP171 (Supplementary Figure 6). The COGs category “Intracellular trafficking, secretion, and vesicular transport (U)” encompassed 61 gene clusters, represented mainly by type VI and type II secretion systems, along with diverse membrane-associated components (Supplementary Figure 7). Genes related to the type VI secretion system were also found within shell- and species-specific genomes, indicating the potential existence of shared and unique system loci. Interestingly, ten gene families associated with the type IV secretion system were primarily identified as species-specific genes in *E. hormaechei* subsp. *steigerwaltii* (Bd_23A), which included 24 identified genes.

### 3.5 Identification and classification of T6SS clusters in *Enterobacter* isolates

**FIGURE 6:**
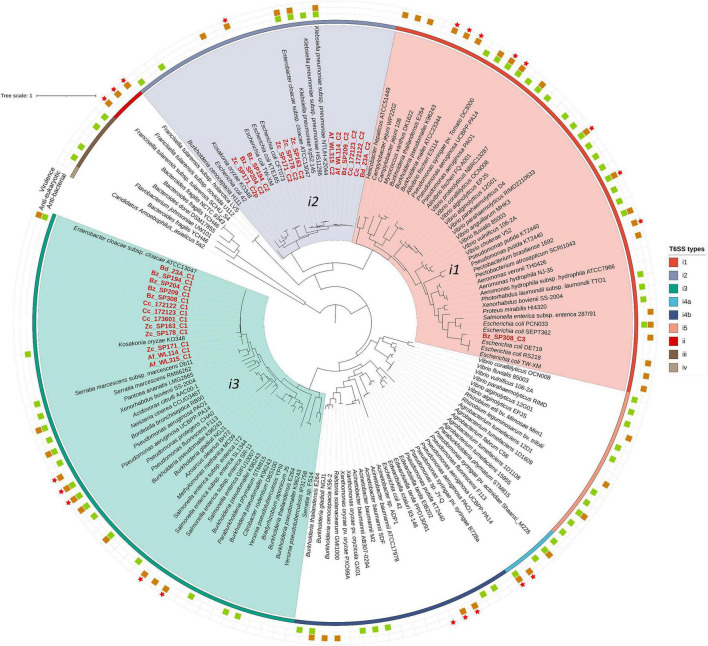
Phylogenetic relationships of T6SS clusters identified in *Enterobacter* strains. A tree was constructed based on the amino acid sequences of TssB using the T6SS classification tool of the SecReT6 web platform. Reference T6SS functions were classified based on SecReT6. Virulence (red star) denotes systems previously shown to induce host virulence via T6SS. Anti-eukaryotic strains (amber rectangle) interact with the host; however, their virulence has not yet been demonstrated. Antibacterial strains (green rectangles) contribute to the bacterial interactions. Tree visualization was performed on ITOL online tool.

Whole-genome sequencing of the *Enterobacter* strains isolated in this study revealed multiple copies of 13 core components (TssA to TssM) of the type VI secretion system (T6SS) in all isolates (Supplementary Table 6). This suggested the existence of multiple distinct T6SS gene clusters within their genomes. T6SS cluster prediction using MacSyFinder indicated that the sequenced genomes encoded at least one putative T6SS cluster. Based on the core components, three distinct T6SS gene clusters were identified within the 13 *Enterobacter* isolates and designated as T6SS_C1, T6SS_C2, and T6SS_C3 (Supplementary Table 7). The analysis of the TssB proteins revealed that T6SS-C1 belonged to subtype i3, T6SS-C2 belonged to subtype i2, and T6SS-C3 belonged to subtype i1 (Figure 6).

**FIGURE 7:**
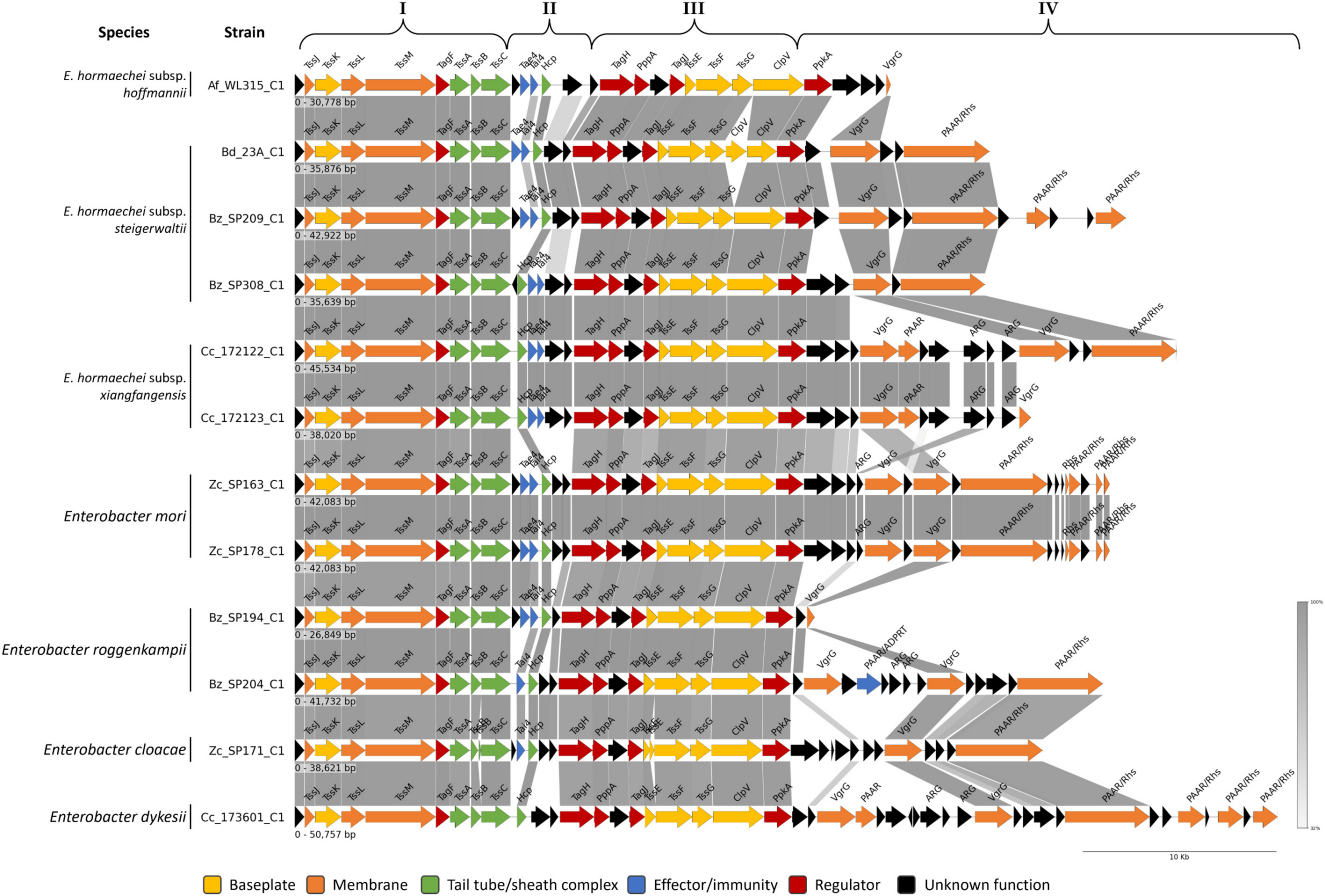
Comparative genomic analysis of regions encoding T6SS_C1 in *Enterobacter* strains. Genes are plotted as arrows, proportional to their lengths, and arranged according to their genomic positions. Genes were grouped according to the method described by Zoued et al. (2014).

The T6SS_C1 cluster was identified in all strains, with a cluster size ranging from 26,849 bp (Bz_SP194, *E. roggenkampii*) to 50,757 bp (Cc_173601, *E. dykesii*) (Supplementary Table 7). An exception was observed for Af_WL114, where an incomplete cluster was detected (seven genes out of 13 genes), which is likely due to the high fragmentation of its genome assembly. Overall, the T6SS_C1 clusters appeared to be complete, as they included 13 core components, regulatory proteins (TagF, TagH, TagJ, and kinase-phosphatase pair PpkA-PppA), and peptidoglycan-targeting effectors/immunity components (Tae4/Tai4), suggesting a potential functionality (Figure 7). The conserved genes were arranged in two syntenic blocks (Block I and Block III) separated by a variable region (Block II) that typically contained Hcp (TssD) and effector/immunity gene pairs (Figure 7). Interestingly, T6SS_C1 in *E. roggenkampii* (Bz_SP204) and *E. cloacae* (Zc_SP171) appeared to encode a predicted immunity protein (Tai4) with no apparent corresponding effector (Tae4). Unlike other strains, *E. dykesii* (Cc_173601) did not encode any effector/immunity pair within the variable region (Block II). An additional variable region (block IV) was located at the end of the cluster adjacent to VgrG (TssI protein) (Figure 7). This variable region harbors components encoding the PAAR domain (sharp conical extension on the VgrG spike), which is generally fused to rearrangement hotspot proteins (Rhs). PAAR/Rhs protein appears within block IV as a single copy in certain strains, such as *E. hormaechei subsp. steigerwaltii* (Bd_23A and Bz_SP308), *E. hormaechei* subsp. *xiangfangensis* (Cc_172123), and *E. cloacae* (Zc_SP171) or multiple copies in strains, such as *E. roggenkampii* (Bz_SP204), *E. dykesii* (Cc_173601), and *E. mori* (Zc_SP178). However, *E. roggenkampii* (Bz_SP194) and *E. hormaechei* subsp. *hoffmannii* (Af_WL315) contained fewer genes in the variable region (block IV), which could be attributed to the position of the T6SS cluster at the end of the assembled contig. Interestingly, *E. roggenkampii* (Bz_SP204) encodes a gene containing an N-terminal PAAR motif and C-terminal toxin domain assigned by RAST annotation as an actin-ADP-ribosylating toxin protein (ADPRT), which is likely exported by the T6SS_C1 system (Figure 7).

**FIGURE 8:**
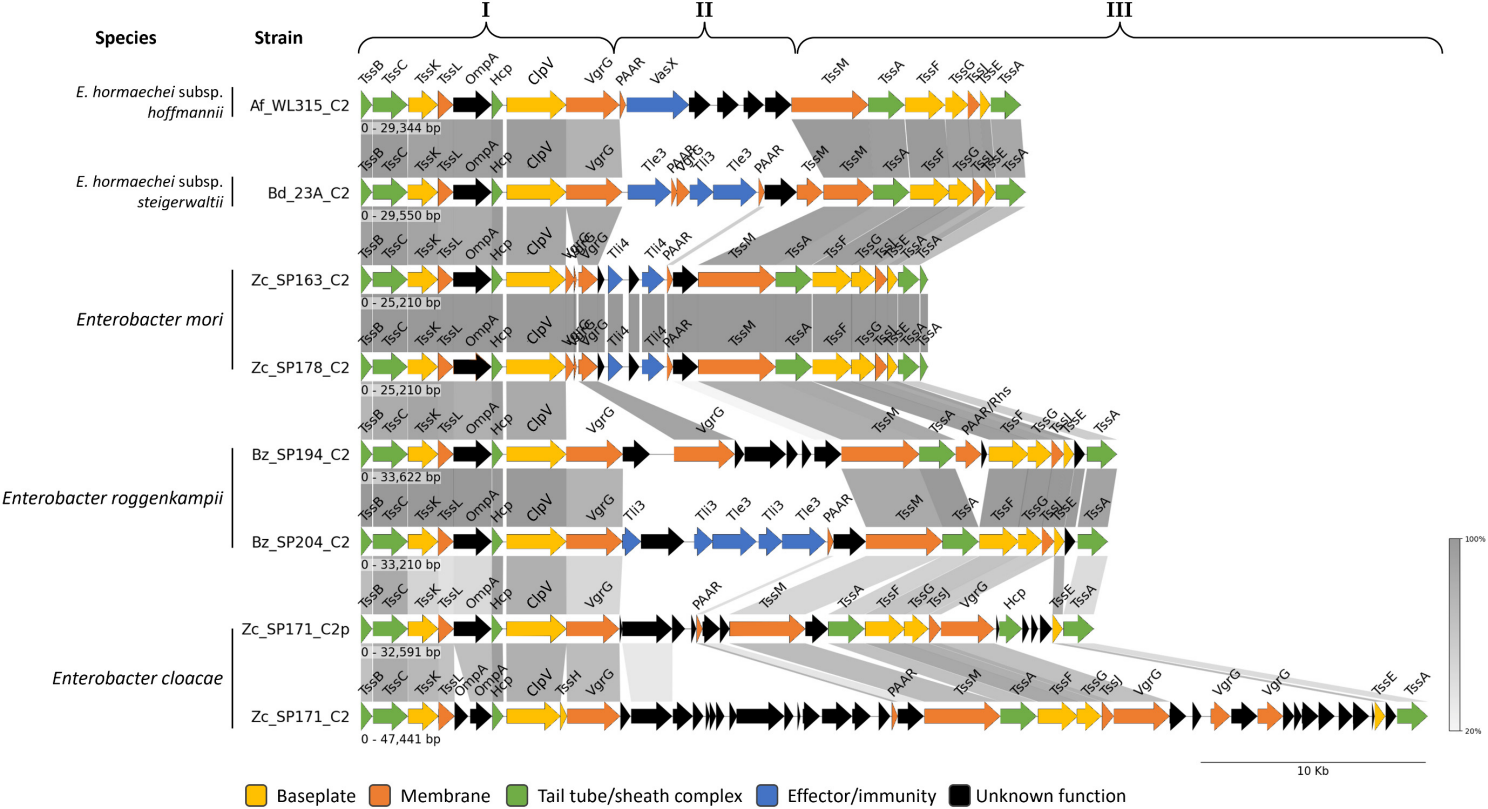
Comparative genomic analysis of regions encoding T6SS_C2 in *Enterobacter* strains. Genes are plotted as arrows, proportional to their lengths, and arranged according to their genomic positions. Genes were grouped according to the method described by Zoued et al. (2014).

The T6SS_C2 cluster was identified in seven *Enterobacter* strains: *E. hormaechei* subsp. *hoffmannii* (Af_WL315), *E. hormaechei* subsp. *steigerwaltii* (Bd_23A), *E. roggenkampii* (Bz_SP194 and Bz_SP204), *E. mori* (Zc_SP163 and Zc_SP178), and *E. cloacae* (Zc_SP171) (Figure 8 and Supplementary Table 7). The size of the clusters ranged from 25,210 bp (in Zc_SP163 and Zc_SP178) to 47,441 bp (in Zc_SP171). In contrast to T6SS_C1, T6SS_C2 clusters lacked genes encoding regulators and showed a different gene arrangement within the conserved and the variable regions (Figure 8). Within T6SS_C2, the variable region (block II) was enriched with effector/immunity gene, PAAR, and VgrG proteins. T6SS lipase effector/immunity pairs (Tle3/Tli3) were identified in *E. hormaechei* subsp. *steigerwaltii* (Bd_23A) and *E. roggenkampii* (Bz_SP204) (Figure 8). In contrast, both strains of *E. mori* (Zc_SP163 and Zc_SP178) encoded only the immunity protein Tli4, without its corresponding effector (Tle4). Additionally, a virulence-associated secretion protein (VasX) was found exclusively in the T6SS_C2 of the strain *E. hormaechei* subsp. *hoffmannii* (Af_WL315). Interestingly, two copies of T6SS_C2 were identified in Zc_SP171 (*E. cloacae*): one located on the plasmid and the other on the chromosome. Although chromosomal T6SS_C2 shares, to some extent, the general genetic organization, it includes additional unknown genes within the variable region in block II. Additionally, Block III included multiple copies of the VgrG gene associated with various uncharacterized genes (Figure 8).

**FIGURE 9:**
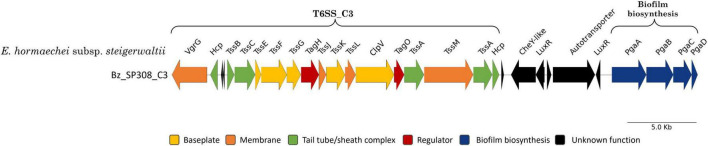
Regions encoding T6SS_C3 in *Enterobacter* strain Bz_SP308. Genes are plotted as arrows, proportional to their lengths, and arranged according to their genomic positions. Genes were grouped according to the method described by Zoued et al. (2014).

The T6SS_C3 cluster, which belongs to subtype i1, has been uniquely identified in *E. hormaechei subsp. steigerwaltii* (Bz_SP308). Notably, this strain lacks the T6SS_C2 cluster (subtype i2), which is present in other closely related strains such as *E. hormaechei subsp. steigerwaltii* (Bd_23A). The length of the T6SS_C3 cluster was approximately 23,579 bp, and it included all 13 core T6SS genes (Figure 9 and Supplementary Table 7). Interestingly, these genes are organized differently than their homologs in the T6SS_C1 and T6SS_C2 clusters, suggesting a potential variation in cluster function. Furthermore, the T6SS_C3 cluster included accessory genes TagH and TagO, while no effector/immunity gene pairs and PAAR genes were characterized within the cluster (Figure 9). Downstream of the T6SS_C3 cluster, strain Bz_SP308 encodes the complete operon pgaABCD responsible for producing poly-β-1,6-N-acetylglucosamine (PNAG), which is essential for biofilm formation (Figure 9).

## 4 Discussion

*Enterobacter* spp. are widely distributed across various fruit fly species and play an important role in host fitness and development. Culture-independent studies based on 16S rRNA gene sequencing have consistently detected *Enterobacter* spp. across developmental stages, host species, and environmental factors, suggesting a stable and ecologically relevant association (Augustinos et al., 2015; Nikolouli et al., 2020; Salgueiro et al., 2022). Although shotgun metagenomics and metagenome assembled genomes offer higher taxonomic resolution than the 16S rRNA gene amplicon approach (Comeault et al., 2025), their routine use is limited by the cost, complexity of the community, and host DNA interference. In addition, metagenomic data alone cannot support direct experimental validation of microbial functions or host-microbial interactions *in vitro* or *in vivo*. Consistent with these findings, our culture-dependent isolation of *Enterobacter* spp. across multiple fruit flies provided strain- and species-level resolution and functional characterization that is unattainable through culture-independent-based approaches. Previous studies have demonstrated that *Enterobacter* strains can influence the host’s fitness and development (Kyritsis et al., 2019; Haytham et al., 2024). However, no systematic analysis has been conducted at the species level to examine the distribution of *Enterobacter* spp. across fruit fly species and the mechanisms by which different species interact with their hosts and other microbiome members. To our knowledge, this is the first study to systematically and comprehensively investigate the distribution of *Enterobacter* species isolated from different fruit flies and explore their potential interactions with hosts and the surrounding environment using a comparative genomic approach.

### 4.1 Prevalence of *Enterobacter hormaechei* across fruit flies and potential coevolution patterns

Previous studies have identified various strains affiliated with the genus *Enterobacter* spp. from fruit fly hosts (Hadapad et al., 2016; Nikolouli et al., 2020; Noman et al., 2021; Salgueiro et al., 2022); however, precise species-level characterization has remained ambiguous because of the high sequence similarity in conserved markers of the 16S rRNA gene, which is commonly used for bacterial identification. In this study, a culture-based approach followed by WGS allowed for the correct delineation of the members of this genus. *E. hormaechei* was consistently present across different fruit fly species. Although few studies have previously reported the isolation of this species from fruit flies, including *A. fraterculus*, *C. capitata*, and *Z. cucurbitae* (Augustinos et al., 2015; Hadapad et al., 2016; Salgueiro et al., 2025), its effects on host fitness and physiology have been extensively studied (Kyritsis et al., 2019; Zhang Q. et al., 2021; Haytham et al., 2024; Yin et al., 2024). Additionally, bioassay tests showed that *E. hormaechei* isolates have an attractive effect on *Z. cucurbitae* and *B. dorsalis* (Wang et al., 2013; Hadapad et al., 2016). Therefore, the persistence of this specific *Enterobacter* species across the examined fruit fly hosts could extrapolate to a comparable influence on the flies used in this study. Thus, these strains could be considered potential new isolates with similar effects.

At the subspecies level, whole-genome-based classification of *Enterobacter* isolates confirmed their taxonomic placement as distinct subspecies, as supported by the ANI and dDDH analyses. The results revealed that *E. hormaechei* strains exhibited considerable genomic variation and clustered into three subspecies: *E. hormaechei* subsp. *steigerwaltii*, *E. hormaechei* subsp. *xiangfangensis*, and *E. hormaechei* subsp. *hoffmannii*. The observed variation in ANI values across the strains classified within *E. hormaechei_B* on GTDB release 220 further suggests ongoing genomic diversification within this lineage. As an additional verification step, taxonomic classification and type strain representation were examined using the updated GTDB release 226. In this release, the previously distinct *E. hormaechei_B* and *E. hormaechei_C* clades were merged, and *E. hormaechei* subsp. *xiangfangensis* LMG27195 was selected as the representative of the clade, while *E. hormaechei* subsp. *steigerwaltii* DSM16691 was assigned as the type strain for the subspecies. The taxonomic reclassification of the strains isolated in this study against the updated GTDB release confirmed their classification to the same *E. hormaechei* subspecies, indicating that subspecies classification remains consistent across GTDB releases, despite the updates in database organization and nomenclature.

Further analysis at the subspecies level revealed a potential host-specific distribution for certain subspecies, such as *E*. *hormaechei* subsp. *hoffmannii* in *Anastrepha* and *E*. *hormaechei* subsp. *xianfangensis* in *Ceratitis*. Conversely, *E*. *hormaechei* subsp. *steigerwaltii* occurred in two closely related *Bactrocera* species. The existence of different *E. hormaechei* subspecies could be due to flies originating from different populations or the adaptation of certain genetic variants to specific hosts. To test the potential evolutionary association between *E. hormaechei* subspecies and their fruit fly hosts, congruence between host and *E. hormaechei* phylogenies was conducted using topology/distance-based methods (Legendre et al., 2002; Balbuena et al., 2013). Although the results demonstrated the coevolution of the fruit fly host and *E. hormaechei* at the subspecies level to some extent, they do not conclusively explain the type of speciation (de Vienne et al., 2013). Host-symbiont association can be a combination of cospeciation and host switching (Kikuchi et al., 2009; Mazzon et al., 2010; de Vienne et al., 2013). For the strains used in the present study, both ParaFit and PACo suggested the presence of global coevolution between the fruit flies and *E. hormaechei* subspecies. However, while PACo demonstrated a specific association between *E. hormaechei* subsp. *xiangfangensis* and its host, *C. capitata*, this association did not align with a cospeciation model when Parafit was used. This difference suggests that other independent events, such as host-switching, may have occurred, reflecting the dynamic nature of the host-symbiont relationship. Since the hosts can acquire or lose symbionts in response to environmental factors, this process results in a complex mosaic of evolutionary histories (Bright and Bulgheresi, 2010; Morrow et al., 2014; Villa et al., 2023). Our hypothesis of fruit fly and *E. hormaechei* coevolution was partially verified; however, more genomic datasets of *Enterobacter* strains from fruit flies are required. Additional genomic datasets will provide deeper insight and further assist in deciphering the relative contributions of cospeciation and/or host switching in shaping the evolutionary trajectories of these symbiotic partners.

### 4.2 Additional *Enterobacter* spp. in fruit flies

In addition to *E. hormaechei*, this study also identified *Enterobacter* species associated with fruit flies, including *E. cloacae* strain isolated from *Z. cucurbitae*. *Enterobacter cloacae* strains are commonly found in the guts of various fruit fly species, including *A. fraterculus* (Salgueiro et al., 2025), *A. ludens* (Kuzina et al., 2001), *B. cacuminata* (Thaochan et al., 2010), *B. dorsalis* (Duan et al., 2024), *B. tryoni* (Thaochan et al., 2010), *B. zonata* (Reddy et al., 2014), and *Z. cucurbitae* (Narit and Anuchit, 2011), and their presence is mostly related to the attractive effect of their host (Wang et al., 2013; Reddy et al., 2014; Duan et al., 2024). Given that only a selected set of strains underwent genome sequencing in this study, it is likely that additional *E. cloacae* strains may also be associated with other fruit fly species examined. Other *Enterobacter* species were also isolated in this study, including *E. mori* isolated from *Z. cucurbitae*, *E. roggenkampii* from *B. zonata*, and *E. dykesii* from *C. capitata*. *Enterobacter mori* was initially identified as the causative agent of bacterial wilt in mulberry (Zhu et al., 2011). It has since been associated with diseases in other plants, including kiwifruit, watermelon, and tomato (Zhang M. et al., 2021; Wu et al., 2023; Ning et al., 2024). In fruit flies, *E. mori* has been isolated from adult *Drosophila melanogaster* (McMullen et al., 2021) and *Zeugodacus tau* (Noman et al., 2021). However, their specific roles in insect hosts remain unclear. *Enterobacter roggenkampii* was first identified as an opportunistic pathogen in clinical settings as part of the *Enterobacter cloacae* complex (Hoffmann and Roggenkamp, 2003). Beyond its clinical relevance, some strains of *E. roggenkampii* function as endophytic diazotrophs, facilitating biological nitrogen fixation in certain plants, including sugarcane and hop-headed barleria (Guo et al., 2020; Kumar and Dubey, 2022). It has also been linked to pathogenicity in other plants, such as mulberry and tomato (Zhou et al., 2021; Guo et al., 2023), as well as in a wide range of hosts, including humans (Wang et al., 2020), fish (Li and Sun, 2024), chickens (Lei et al., 2020), and dogs (Sato et al., 2021). *Enterobacter dykesii* is a newly identified species initially isolated from bean sprouts. It harbors potentially transferable antibiotic resistance genes, which may contribute to their spread through the food chain (Cho et al., 2021). The present study marks the first documented occurrence of *E. roggenkampii* and *E. dykesii* in fruit flies, specifically within laboratory populations of *B. zonata* and *C. capitata*, respectively. Identification of these *Enterobacter* species across different fruit fly hosts highlights the complex and varied relationships between fruit flies and their associated microbial communities. Further investigations are required to understand their functional roles and to assess their potential applications in improving fitness or as attractants.

### 4.3 Pangenome structure revealed the diversity of *Enterobacter* species

Pangenome analysis is a useful approach to uncover the genetic diversity and evolutionary dynamics of bacterial species (Brockhurst et al., 2019). The core genome is fundamental to the basic lifestyle of bacteria, whereas the shell and species-specific genomes contribute to species diversity, environmental adaptability, and other distinct traits (Vernikos et al., 2015; Costa et al., 2020). The pangenome analysis conducted in this study revealed a dynamic genetic landscape among *Enterobacter* species, with significant differences in the core, shell, and species-specific genome composition. The high proportion of metabolism-related gene clusters in the core genome suggests their conserved role in essential biological functions. The shell- and species-specific genomes also contain a significant number of metabolism-related genes, which primarily encode proteins with functions similar to those found in the core genome. Proteins with similar functions often share structural domains; however, their sequence identity may remain too low to cluster into the same group, even when the sequence similarity threshold is reduced.

In the species-specific genome, the analysis highlighted the significant genomic plasticity of the strains studied, which is largely driven by the presence of mobile genetic elements, such as prophages and transposons. These elements may facilitate adaptation to diverse environmental conditions, including host-specific niches, by mediating horizontal gene transfer (Darmon and Leach, 2014). Through this process, mobile genetic elements can carry various genes, including those responsible for antimicrobial and metal resistance, virulence, and catabolic functions (Darmon and Leach, 2014; Tokuda and Shintani, 2024). Variations in functional gene content were also observed across strains, particularly in different COG categories. Notably, *E. hormaechei* subsp. *steigerwaltii* strains Bd_23A and Bz_SP209 exhibited additional flagella-related genes, suggesting the presence of a secondary flagellar locus. This feature is uncommon within the *genus Enterobacter* and may provide functional advantages (De Maayer et al., 2020a). De Maayer et al. suggested that such secondary flagellar loci may serve multiple roles, including cell motility, host colonization ability, and putative secretory functions (De Maayer et al., 2020a,2020b). Therefore, the presence of a secondary flagellar system in strains Bd_23A and Bz_209 may confer adaptive advantages, enhancing their ability to colonize diverse niches or interact with their host. Further investigations are required to confirm the presence of this system and to elucidate its functional roles within *Enterobacter* strains associated with fruit flies. Additionally, the presence of CRISPR-Cas components and restriction-modification systems, mainly in *E. hormaechei* subsp. *steigerwaltii* (Bd_23A and Bz_SP308), *E. roggenkampii* (Bz_SP204 and Bz_SP194), and *E. cloacae* (Zc_SP171), highlights the role of these defense mechanisms in bacterial survival. These systems are known to provide adaptive and hereditary immunity against previously encountered bacteriophages and plasmids, reinforcing genome stability, and protection against foreign genetic elements (Oost et al., 2009; Terns and Terns, 2011). Interestingly, the absence of genes related to CRISPR-Cas systems in some *Enterobacter* strains suggests that alternative defense strategies may be at play, which should be explored in future studies. Overall, while the present pangenome analysis underscores the genomic diversity between *Enterobacter* species isolated from fruit flies, it is limited by the relatively low number of genomes analyzed. A larger dataset is necessary to capture the extent of genomic variation and adaptive strategies across *Enterobacter* species in fruit fly populations.

### 4.4 Type VI secretion systems and their role in bacterial interaction

The role of T6SS in competition and pathogenicity has been demonstrated in many *Enterobacter* clinical isolates (Soria-Bustos et al., 2020; Anderson et al., 2023; Peng et al., 2024). However, the role of the T6SS in insect-associated *Enterobacter* isolates remains unclear. Genomic analysis of *Enterobacter* spp. isolated from fruit flies in this study revealed the presence of three distinct T6SS clusters, designated as T6SS_C1, T6SS_C2, and T6SS_C3. The genetic organization of these clusters appears to be complete, with all the conserved core genes required for a functional T6SS. T6SS subtypes were identified based on TssB proteins: T6SS_C1 (subtype i3), T6SS_C2 (subtype i2), and T6SS_C3 (subtype i1), suggesting functional specialization among these systems (Schwarz et al., 2010; Soria-Bustos et al., 2020).

T6SS_C1 (subtype i3) was consistently present across all analyzed strains with a highly conserved structural gene organization, indicating its potential role as a core T6SS system in these *Enterobacter* isolates. However, considerable variation has been observed in arrays that encode effectors and genes with unknown functions. Among the identified effectors, the antibacterial peptidoglycan amidase effector, Tae4, was detected. Tae4 hydrolyzes the acceptor stem peptide bond between γ-d-Glu and meso-diaminopimelic acid (mDAP), leading to cell wall degradation (Russell et al., 2012; Zhang et al., 2013). To prevent self-intoxication, bacteria encode the immunity protein Tai4, which neutralizes Tae4 activity (Zhang et al., 2013). The presence of Tae4/Tai4 pair within the T6SS_C1 cluster in the analyzed *E. hormaechei* strains (Af_WL315, Bd_23A, Bz_SP209, Bz_SP308, Cc_172122, and Cc_172123), both *E. mori* strains (Zc_SP163 and Zc_SP178), and one strain of *E. roggenkampii* (Bz_SP194) suggested that these strains actively engage in competitive interactions, aiding the host colonization (Sana et al., 2016). Conversely, the *E. roggenkampii* (Bz_SP204) and *E. cloacae* (Zc_SP171) strains appeared to have lost the Tae4 effector gene but retained the Tai4 immunity gene, which may provide them with immunity against Tae4-producing competitors without investing in active antagonism. Additionally, PAAR/actin-ADP-ribosylating toxin protein was identified exclusively in the *E. roggenkampii* (Bz_SP204) strain. These toxins have been reported to play dual roles in bacterial competition and host interactions. For instance, *Aeromonas hydrophila* utilizes T6SS to secrete VgrG1 effector protein, which possesses actin-ADP-ribosylating activity. This effector disrupts the host actin cytoskeleton and induces toxicity (Suarez et al., 2010). In contrast, certain bacteria utilize ADP-ribosylating toxins in the T6SS-mediated interbacterial competition (Ting et al., 2018; Bullen et al., 2022). For example, *Serratia proteamaculans* delivers a T6SS toxin that ADP-ribosylates the essential bacterial protein FtsZ in target bacteria, thereby inhibiting cell division (Ting et al., 2018). Similarly, *Pseudomonas aeruginosa* was reported to secrete an ADP-ribosyltransferase toxin via T6SS, which modifies non-coding RNAs in competitor cells, leading to the inhibition of multiple essential cellular processes and ultimately, cell death (Bullen et al., 2022). Therefore, the association of the actin-ADP-ribosylating toxin with a PAAR domain within the T6SS_C1 cluster in the *E. roggenkampii* Bz_SP204 strain indicates its potentially active role in host interactions and/or bacterial competition. However, further experiments are required to assess the expression of this potential effector and the factors that influence its activity.

T6SS_C2 (subtype i2) was found in most of the strains analyzed in this study, which is consistent with previous reports on this cluster in *Enterobacter* species (Soria-Bustos et al., 2020; Anderson et al., 2023; Peng et al., 2024). The absence of this cluster in certain *E. hormaechei* strains (Af_WL114, Bz_SP209, Cc_172122, and Cc_172123) may be attributed to incomplete or fragmented genome assemblies, which could have hindered the detection of the full cluster. Additionally, the presence of at least two copies of T6SS core genes in these strains suggests that the T6SS_C2 cluster is likely present but not fully assembled in the current genomic data. A complete genome assembly would be able to confirm its presence. Unlike T6SS_C1, T6SS_C2 in *E. hormaechei* subsp. *steigerwaltii* (Bd_23A) and *E. roggenkampii* (Bz_SP204) encode T6SS lipase effectors (Tle3), which play a critical role in interbacterial competition. The Tle effectors function by hydrolyzing phospholipids in the membranes of competing bacteria, ultimately leading to cell lysis (Berni et al., 2019; Jurënas and Journet, 2021). Virulence-associated secretion protein X (VasX) encoded within T6SS_C2 of *E. hormaechei* subsp. *hoffmannii* (Af_WL315) has been implicated in T6SS-mediated virulence in *Vibrio cholerae* (Dong et al., 2013). In Af_WL315, VasX appears to be non-functional due to the absence of the transcriptional activator VasH, which is essential for its expression (Monjarás Feria and Valvano, 2020).

Additionally, the strain *E. hormaechei* subsp. *steigerwaltii* (Bz_SP308) lacks genetic evidence to encode T6SS_C2; instead, it encodes a T6SS_C3 cluster belonging to the T6SS subtype i1. Notably, this cluster is characterized by the absence of genes encoding effector and PAAR proteins, which are essential for effector delivery and the structural stabilization of the T6SS apparatus (Burkinshaw et al., 2018; Dyrma et al., 2025). Given the critical role of PAAR proteins in sharpening the secretion system spike and facilitating effector secretion, the lack of these components raises questions regarding the functional capacity of T6SS_C3 beyond basic secretion. The absence of effectors suggests that this cluster does not participate in direct interbacterial or host interactions. Instead, T6SS_C3 may play a role in bacterial environment interactions, such as biofilm formation (Chen et al., 2020; Lin et al., 2021), given its genomic proximity to the pgaABCD operon, which is involved in biofilm exopolysaccharide synthesis. Further experimental validation is needed to assess the expression and activity of T6SS_C3 and to determine whether it has a specialized role in biofilm formation or other non-canonical T6SS functions.

In summary, the presence of two distinct T6SS clusters in *Enterobacter* isolates suggests that both systems are functional within their respective hosts. However, variations in the genetic organization of each cluster, together with differences in the encoded effectors and their respective delivery mechanisms, may indicate distinct ecological roles such as bacterial competition or other interactions with specific host environments. These variations likely reflect selective pressures that shape T6SS functionality across different strains. Large-scale metagenomic and metatranscriptomic studies would further enrich this framework. Such studies would assess the T6SS clusters’ prevalence, structural diversity, and whether they are actively expressed *in situ* across multiple *Enterobacter* strains, as well as their activity in relation to the host and other members of the fruit fly’s symbiotic community.

## 5 Conclusion

This study presents a comprehensive genomic characterization of *Enterobacter* strains, offering new insights into their taxonomy, evolutionary relationships, and functional diversity. In addition, it highlights the potential role of T6SS in the symbiosis between these bacteria and their fruit fly hosts. The widespread occurrence of *E. hormaechei* and its subspecies across different hosts suggests their potential for coevolution and strain-specific adaptations. Genome-wide comparisons revealed a variable pangenome structure and the presence of three distinct T6SS types, with T6SS-C1 and T6SS-C2 being widely distributed, whereas T6SS-C3 was exclusively found in *E. hormaechei* subsp. *steigerwaltii* (Bz_SP308) from *B. zonata*. The coexistence of multiple *Enterobacter* species/subspecies, along with the presence of multiple T6SS types within closely related flies of the Tephritidae family, suggests their potential involvement in interspecific and/or intraspecific bacterial competition and potential environmental adaptation. Future research should focus on the experimental validation of T6SS expression, function, and ecological roles as well as further exploration of host-specific adaptations and their impact on fruit fly fitness and behavior. Refining *Enterobacter* taxonomy through expanded genomic datasets and functional studies is essential for resolving classification discrepancies and improving our understanding of bacterial evolution and host associations. Expanding our knowledge of *Enterobacter*-host interactions will contribute to the development of novel pest management strategies that exploit symbiotic relationships, such as microbiome-based biocontrol approaches targeting fruit fly populations.

## Data Availability

The datasets presented in this study can be found in online repositories. The names of the repository/repositories and accession number(s) can be found at: https://www.ncbi.nlm.nih.gov/ PRJNA1093357.

## Footnotes

1
https://blast.ncbi.nlm.nih.gov/Blast.cgi

2
https://github.com/tseemann/shovill

3
https://github.com/nanoporetech/medaka

4
https://tygs.dsmz.de

5
https://www.geneious.com

6
https://github.com/moshi4/COGclassifier

7
https://bioinfo-mml.sjtu.edu.cn/SecReT6/phylogenetic_analysis.php

8
https://github.com/moshi4/pyGenomeViz

## Supplementary material

Supplementary Figure 1 Average Nucleotide Identity (ANI) comparison between *Enterobacter hormaechei* subspecies. The genomes of *Enterobacter* isolates in this study (red) and their closest relatives in the GTDB (green). Type strains for each subspecies, if available, were indicated with an asterisk (*).

Supplementary Figure 2 Distribution of genes in *Enterobacter* spp. pangenomes. **(A)** Upset graph representing core, shared, species/subspecies-specific, and unique (singleton) genes among strains. Only groups sharing more than 10 genes were displayed. **(B)** Total cluster count within each pangenome category (core, shell, and species-specific). Percentages within each bar represent the proportion of gene clusters assigned to COG families vs. unassigned genes. **(C)** Distribution of COG types for core, shell, and species-specific; bar length represents the proportion of each functional gene type in the corresponding category.

Supplementary Figure 3 Normalized count of species-specific COGs functional categories across *Enterobacter* spp.

Supplementary Figure 4 Distribution of COGs functions within “Mobilome: prophages, transposons (X)” category across *Enterobacter* spp. pangenome. The cell numbers represent the number of genes identified in each genome.

Supplementary Figure 5 Distribution of COGs functions within “Cell motility (N)” category across *Enterobacter* spp. pangenome. Cell numbers represent the number of genes identified in each specific genome.

Supplementary Figure 6 Distribution of COGs functions within “Coenzyme transport and metabolism (H)” category across *Enterobacter* spp. pangenome. Cell numbers represent the number of genes identified in each specific genome.

Supplementary Figure 7 Distribution of COGs functions within the “intracellular trafficking, secretion, and vesicular transport (U)” categories across *Enterobacter* spp. pangenomes. The cell numbers represent the number of genes identified in each specific genome.

Supplementary Table 1 List of reference *Enterobacter* genomes.

Supplementary Table 2 Taxonomic classification of all *Enterobacter* isolates based on GTDB database.

Supplementary Table 3 Digital DNA-DNA hybridization (dDDH) for *Enterobacter hormaechei* subspecies strains and their closest type strain material, estimated based on Genome-to-Genome Distance Calculator (GGDC) in TYGS platform. “d4” represents the recommended dDDH formula d4.

Supplementary Table 4 ParaFit and PACo tests of host-*Enterobacter* spp. coevolution.

Supplementary Table 5 Fisher’s exact test for differential COG category presence across pangenomes. *P*-values from Fisher’s exact test comparing the presence of specific COG categories among core, shell, and species-specific pangenome. Significance levels are indicated as follows: **P* < 0.05, ***P* < 0.01, and ****P* < 0.001.

Supplementary Table 6 Prevalence of core T6SS genes across *Enterobacter* strains.

Supplementary Table 7 T6SS clusters identified in the *Enterobacter* strains examined in this study.
