# Supplementary figures and images for: Evolutionary dynamics of type VI secretion systems in fruit fly-associated Enterobacter
Source: Front Microbiol. 2026 Mar 5;17:1755534. doi: 10.3389/fmicb.2026.1755534 (PMC12999792; doi:10.3389/fmicb.2026.1755534)

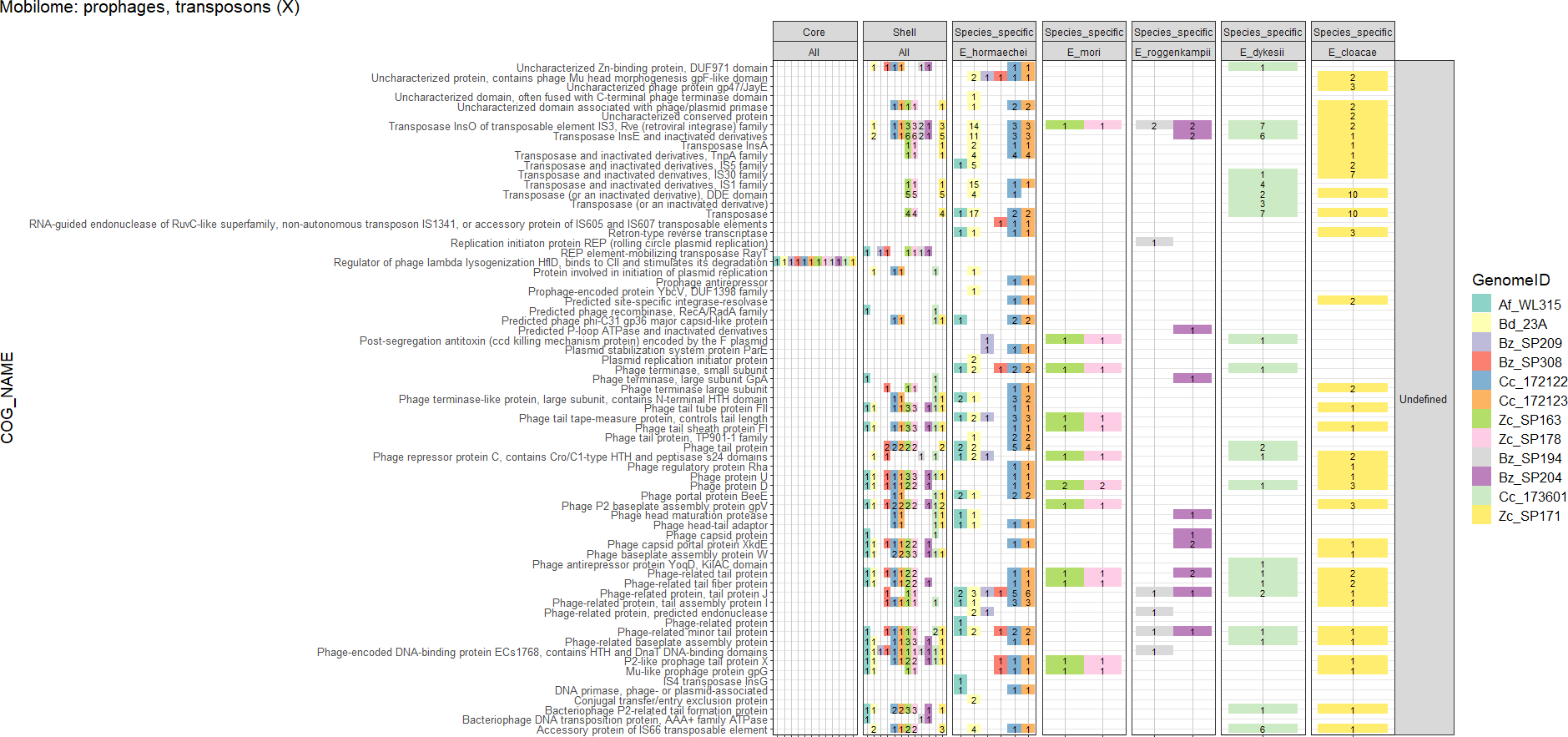

Supplement: Supplementary Figure 4 — Distribution of COGs functions within “Mobilome: prophages, transposons (X)” category across Enterobacter spp. pangenome. The cell numbers represent the number of genes identified in each genome. [file Image_4.tif]

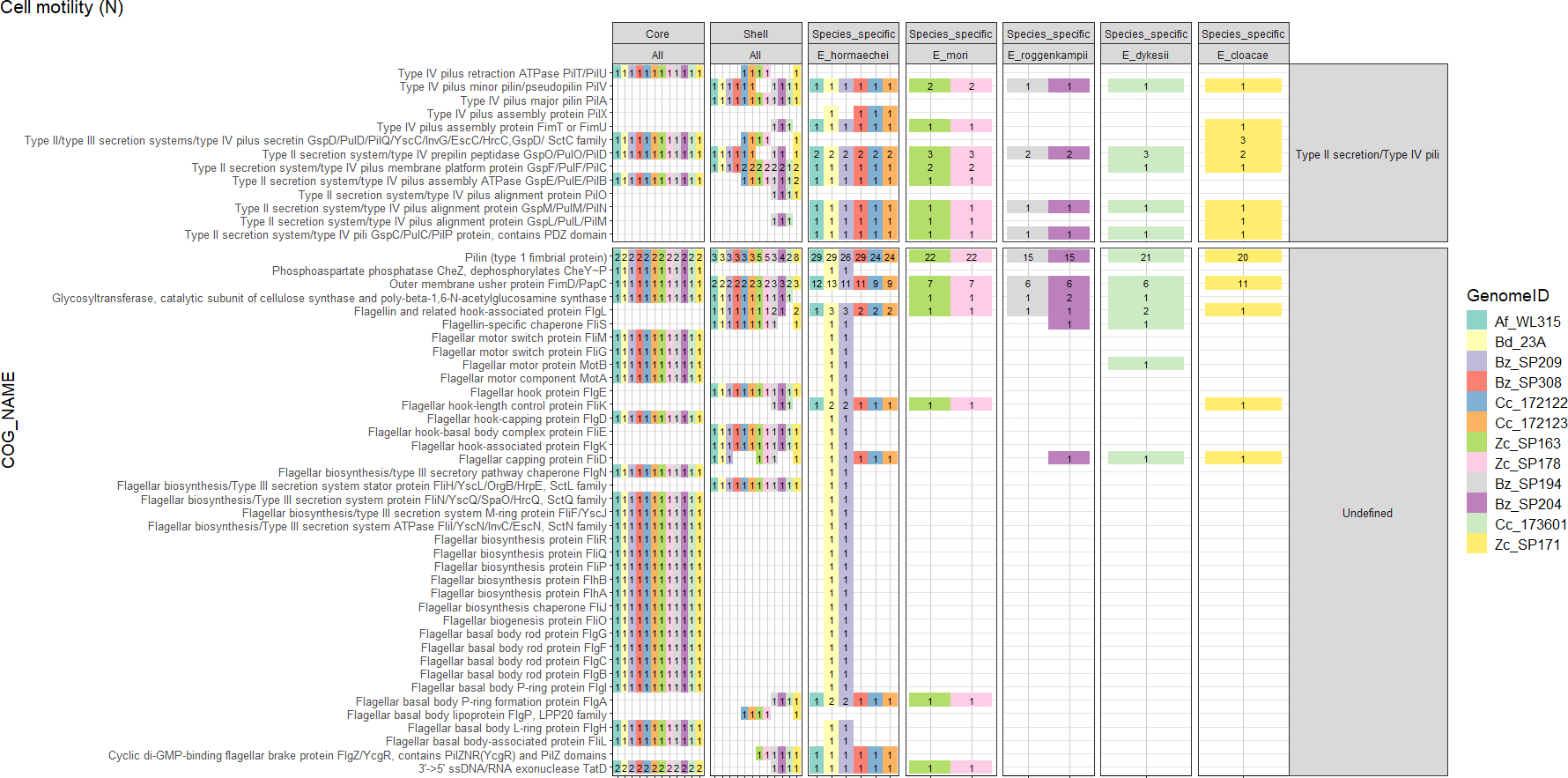

Supplement: Supplementary Figure 5 — Distribution of COGs functions within “Cell motility (N)” category across Enterobacter spp. pangenome. Cell numbers represent the number of genes identified in each specific genome. [file Image_5.tif]

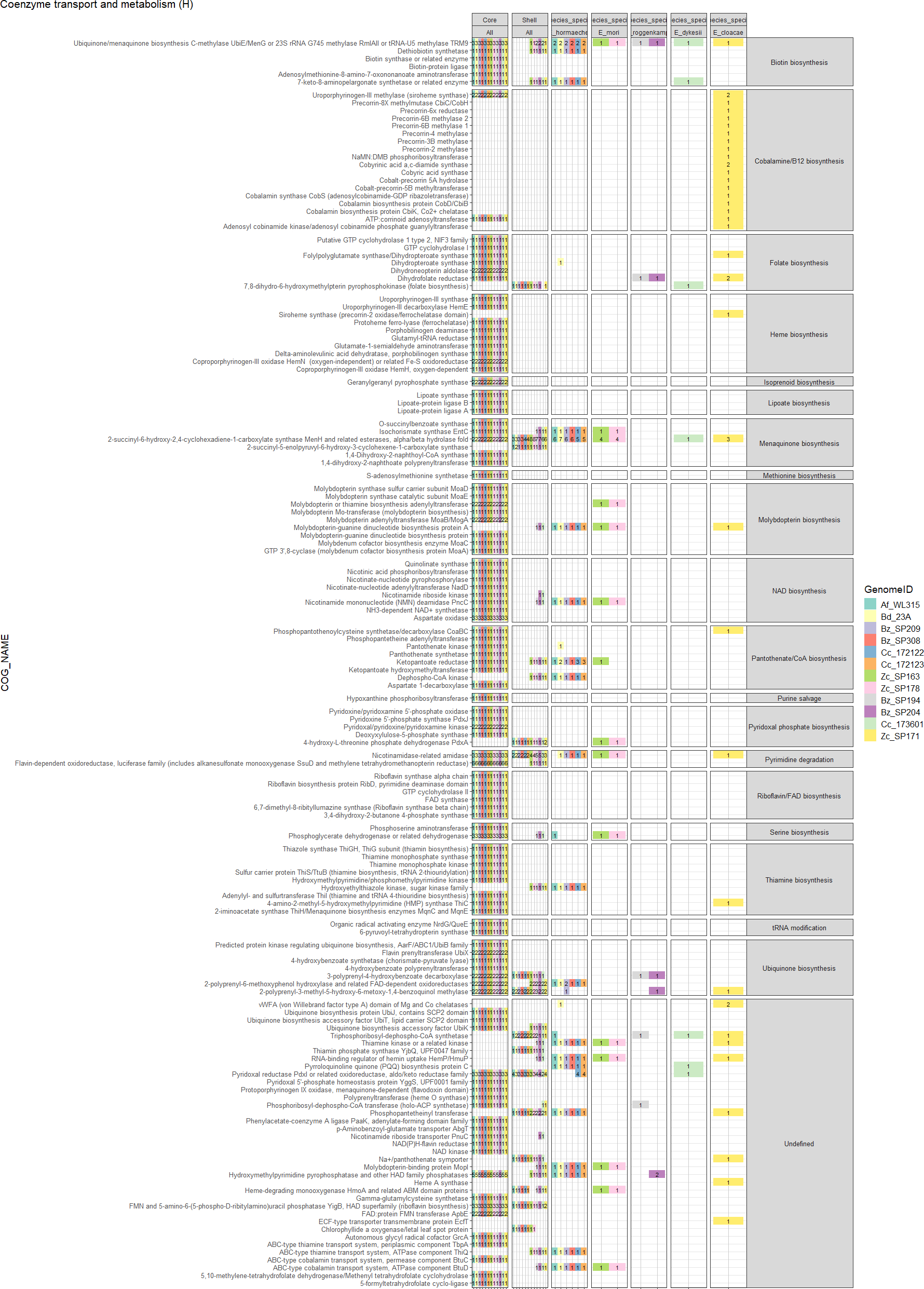

Supplement: Supplementary Figure 6 — Distribution of COGs functions within “Coenzyme transport and metabolism (H)” category across Enterobacter spp. pangenome. Cell numbers represent the number of genes identified in each specific genome. [file Image_6.tif]

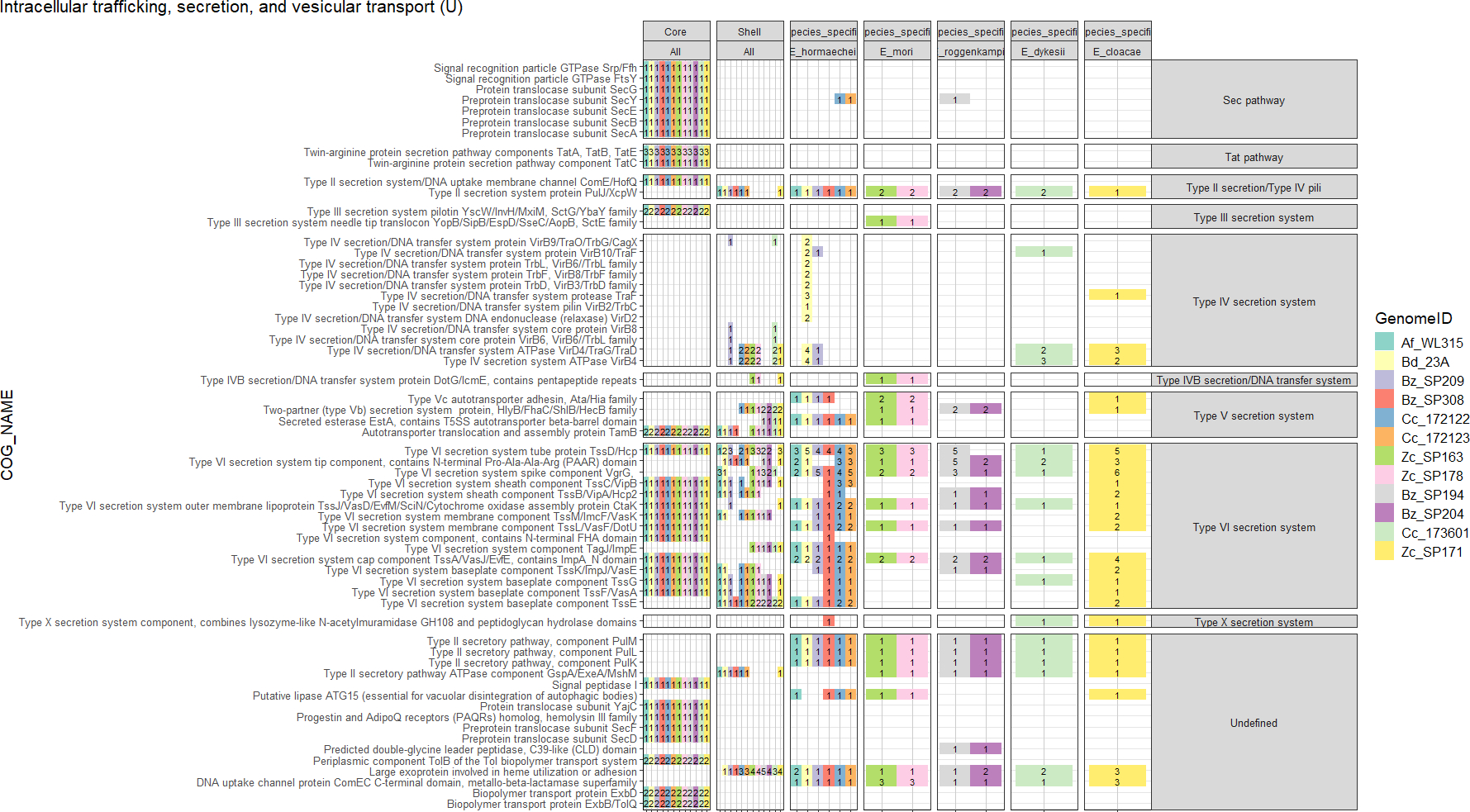

Supplement: Supplementary Figure 7 — Distribution of COGs functions within the “intracellular trafficking, secretion, and vesicular transport (U)” categories across Enterobacter spp. pangenomes. The cell numbers represent the number of genes identified in each specific genome. [file Image_7.tif]
